# Determining the accuracy and suitability of common analytical techniques for sophorolipid biosurfactants

**DOI:** 10.1093/jimb/kuae021

**Published:** 2024-06-21

**Authors:** Benjamin Ingham, Rehana Sung, Phil Kay, Katherine Hollywood, Phavit Wongsirichot, Alistair Veitch, James Winterburn

**Affiliations:** Department of Chemical Engineering, The University of Manchester, Oxford Road, Manchester M13 9PL, UK; Manchester Institute of Biotechnology, Department of Chemistry, University of Manchester, Manchester M1 7DN, UK; JMP Statistical Discovery LLC, Wittington House, Henley Road, Medmenham, Marlow SL7 2EB, UK; Manchester Institute of Biotechnology, Department of Chemistry, University of Manchester, Manchester M1 7DN, UK; Department of Chemical Engineering, The University of Manchester, Oxford Road, Manchester M13 9PL, UK; Holiferm Ltd, Unit 15, Severnside Trading Estate, Textilose Road, Trafford Park, Stretford, Manchester M17 1WA, UK; Department of Chemical Engineering, The University of Manchester, Oxford Road, Manchester M13 9PL, UK

**Keywords:** Sophorolipid, Biosurfactants, Quantification, Gravimetric, High-performance liquid chromatography, Solvent extraction

## Abstract

To determine the performance of a sophorolipid biosurfactant production process, it is important to have accurate and specific analytical techniques in place. Among the most popular are the anthrone assay, gravimetric quantification (hexane:ethyl acetate extraction), and high-performance liquid chromatography (HPLC). The choice of analytical tool varies depending on cost, availability, and ease of use; however, these techniques have never been compared directly against one another. In this work, 75 fermentation broths with varying product/substrate concentrations were comprehensively tested with the 3 techniques and compared. HPLC–ultraviolet detection (198 nm) was capable of quantifying C18:1 subterminal hydroxyl diacetylated lactonic sophorolipid down to a lower limit of 0.3 g/L with low variability (<3.21%). Gravimetric quantification of the broths following liquid:liquid extraction with hexane and ethyl acetate showed some linearity (*R*^2^ = .658) when compared to HPLC but could not quantify lower than 11.06 g/L, even when no sophorolipids were detected in the sample, highlighting the non-specificity of the method to co-extract non-sophorolipid components in the final gravimetric measure. The anthrone assay showed no linearity (*R*^2^ = .129) and was found to cross-react with media components (rapeseed oil, corn steep liquor, glucose), leading to consistent overestimation of sophorolipid concentration. The appearance of poor biomass separation during sample preparation with centrifugation was noted and resolved with a novel sample preparation method with pure ethanol. Extensive analysis and comparisons of the most common sophorolipid quantification techniques are explored and the limitations/advantages are highlighted. The findings provide a guide for scientists to make an informed decision on the suitable quantification tool that meets their needs, exploring all aspects of the analysis process from harvest, sample preparation, and analysis.

## Graphical Abstract

**Graphical Abstract:**
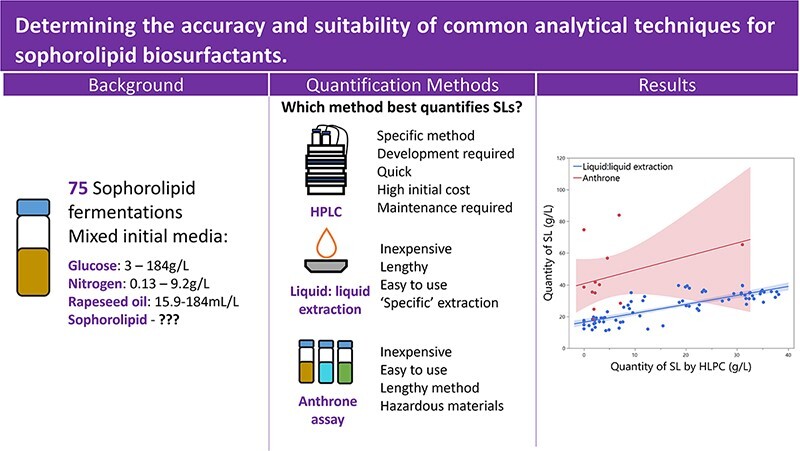

## Introduction

Sophorolipids (SLs) are one of the most highly researched biosurfactants to date, with commercial manufacturing appearing with innovations in process design, strain selection, and feedstock utilization all working towards improving their market availability (Ciesielska et al*.*, 2016; Claus & Van Bogaert, 2017; Dardouri et al*.*, 2021; Dierickx et al*.*, 2022; Ingham & Winterburn, 2022; Kobayashi et al*.*, 2023; Liu et al*.*, 2022). In all these areas, the marker of a productive/non-productive change to the process is determined by an analytical method that aims to quantify the amount of SL present in the fermentation broth. Despite this, the specificity and sensitivity of these analytical methods are under-researched and cross-comparisons have not been performed. Like many biosurfactant classes, SLs can be produced as a variety of structural congeners during the fermentation process, quickly leading to a diverse/complex mixture of analytes that present different physiochemical characteristics and different responses to any given analytical method. Without an understanding of the capabilities/limitations of a given analytical method, it is difficult to say with confidence the form and actual quantity of SL present in the sample being investigated. In production processes, accurate quantification is essential in being able to gauge the productivity of the fermentation to determine the health of a batch and apply corrective changes to boost production/reduce loss. In research, an understanding of which analytical method has been applied and its limitations is important when comparing the impact of one work to another. Often, reviews/comparisons between different SL production processes compare between works that have used completely different analytical techniques, which can skew the impact/importance of any given work (Celligoi et al*.*, 2020; Claus & Van Bogaert, 2017; Wongsirichot et al., 2021). The analytical technique used should be clearly stated and comparison between works should be done carefully when denoting a work as productive/non-productive. In an ideal world, a single, specific method would be used, but this is limited by cost, availability of specific equipment (e.g. high-performance liquid chromatography [HPLC]), and the knowledge/skill sets available in any given research environment. For example, liquid:liquid extraction (LLE) and gravimetric quantification is a simple, easy-to-learn method with minimal costs for solvents, tips, pipettes, and trays, whereas HPLC requires specialist, expensive equipment supported by dedicated staff, with associated upkeep and training to operate.

**Fig. 1.**
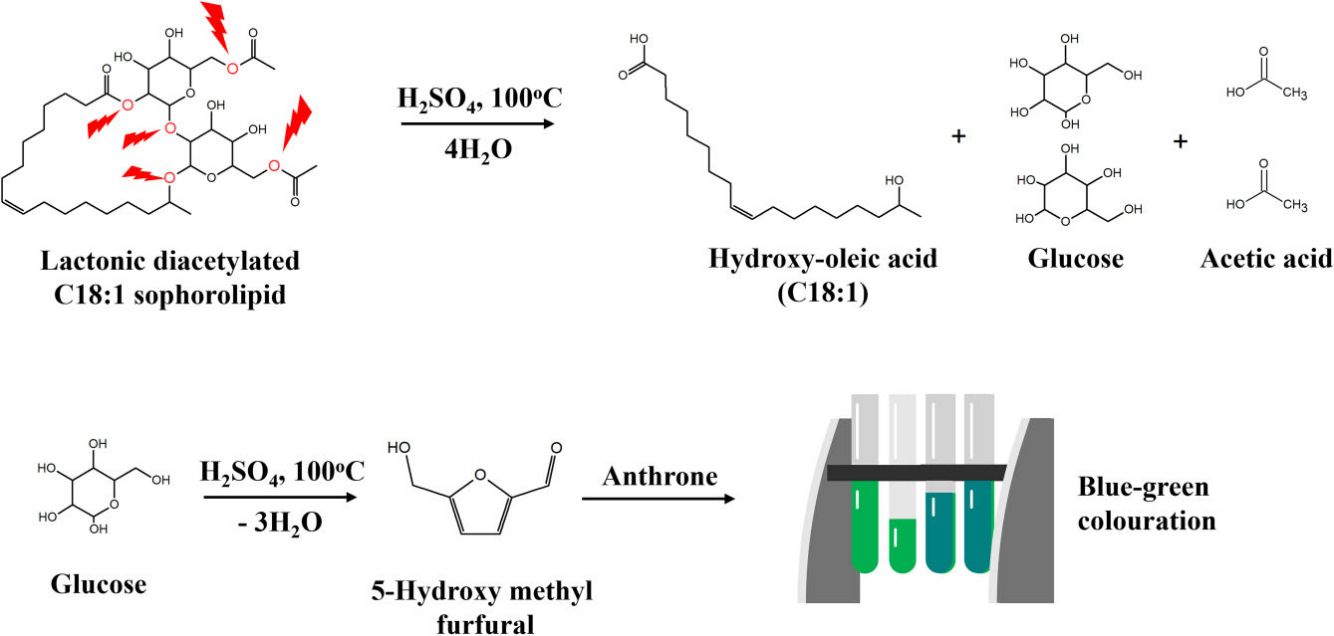
The anthrone assay operates by cleaving ether bonds in the sophorolipid structure (top left, red), releasing the hydroxylated fatty acid (of any length and saturation), acetic acid (in mono- or diacetylated forms), and glucose, which undergoes further hydrolysis to form 5-hydroxymethyl furfural that complexes with anthrone to form a blue–green colour that can be detected at 620 nm.

This work focuses on the three commonly applied quantitative methods for SL biosurfactants: the anthrone assay, LLE, and HPLC. The anthrone assay operates under heated acidic conditions (see Fig. 1), cleaving the ether bonds of the SL structure, releasing glucose, which is further hydrolysed to 5-hydroxymethyl furfural (5-HMF), forming a complex with anthrone with blue–green colouration that can be quantified spectrophotometrically (Chatterjee et al*.*, 2022). LLE is the method most commonly used for SL quantification, utilizing the solubility of rapeseed oil in hexane and SL in ethyl acetate to selectively isolate and quantify (gravimetrically) the product following drying (Ashby et al*.*, 2005; Bajaj & Annapure, 2015; Kim et al*.*, 2020). HPLC utilizes the separation of SL congeners on a chromatographic column, taking advantage of their differing interactions with the stationary and mobile phases to effectively separate congeners that can be subsequently detected with a range of interchangeable detector types, including ultraviolet (LC-UV), mass spectrometry (LC-MS), and evaporative light scattering detection (LC-ELSD) (Bajaj & Annapure, 2015; Davila et al*.*, 1993; Ribeiro et al*.*, 2012; Thaniyavarn et al*.*, 2008). These analytical techniques present different levels of specificity and sensitivity and offer different advantages in terms of cost, turnaround time, accuracy, and ease of use. Direct comparison of these methods on the same samples has never been performed in the literature.

In this work, a comprehensive analysis of the SL content of 75 fermentation samples was quantified using the anthrone assay, LLE, and HPLC–UV, with results compared statistically to understand the suitability of each method for SL quantification. The limitations and advantages of these techniques are discussed and data presented in order to allow scientists to make informed decisions on which method is most suitable depending on the available equipment and required level of understanding. Understanding of these limitations and comparability of methods is important in both academic and industrial settings. As such, the findings will aid in the ongoing research and commercialization efforts.

## Methods and Materials

### Materials

Standards of C18:1 diacetylated lactonic SL were acquired from Biobase Europe (Belgium) and Biosynth (UK). Standards of C18:1 non-acetylated acidic SL were acquired from Biosynth (UK). Ethyl acetate, hexane, anthrone, glucose, and corn steep liquor were acquired from Sigma–Aldrich (Bulgaria). Sulphuric acid, pure ethanol, ammonium sulphate, HPLC-grade water, and acetonitrile (ACN) were acquired from Fisher Scientific (UK). Rapeseed oil was also used (Crisp N’ Dry, UK).

### Fermentation Samples

Fermentation samples from the central composite design work of Ingham and Winterburn (2022) were used in this study to compare the quantitative abilities of different analytical methods (for a full list, see Supplementary Appendix 1). A total of 75 fermentation samples were selected for quantification, representing a range of substrate composition, with variation in glucose, corn steep liquor, ammonium sulphate, and rapeseed oil and SL concentrations.

### HPLC Method Development

#### Sample preparation

To resolve observed issues in the fermentation samples post-centrifugation (weak pelleting, cell debris), differing volumes of pure ethanol were added (1:1 ratio and 2:1 ratio) to fermentation samples, vortexed at 2,000 rpm for 30 s, and centrifuged for 10 min at 4,000 rpm. The final amount of ethanol required was selected based on the formation of a single, clear supernatant free of cell debris.

To determine whether addition of ethanol improved the recovery of SLs from the fermentation broth, a supernatant-only and supernatant/ethanol mixture was prepared in duplicate and filtered with a 0.45-µm syringe filter (Merck, Germany) and tested under the “final” HPLC conditions alongside a C18:1 diacetylated lactonic SL calibration curve (prepared in pure ethanol) and the final concentration quantified.

#### Determination of chromatography conditions for peak resolution

HPLC analysis was performed using an Agilent 1200 HPLC Diode Array Detector system using a Macherey-Nagel™ Nucleosil™ 100 mm × 3 µm × 4.7 mm C18 EC column. The mobile phases were HPLC-grade water and ACN with no additions. Gradient conditions were kept the same at each condition (temperature and flow rate), with a gradient of 30% ACN for 5 min, 30%–72% ACN for 30 min, 72%–100% ACN for 3 min, 100% ACN for 10 min, 100%–30% ACN for 1 min, and equilibration at 30% ACN for 10 min for a total run time of 59 min. The diode array was set to scan from 194–800 nm (1-nm increments), and the best wavelength was selected to provide optimum height of the C18:1 diacetylated ω − 1 lactonic peak.

To find HPLC conditions that allowed for separation of the congeneric forms of SLs (primarily the terminal and subterminal C18:1 diacetylated lactonic form and C18:1 non-acetylated acidic forms), the temperature and flow rate were altered according to Table 1, using 50 g/L C18:1 diacetylated lactonic standard, 10 µl injection volume. After the optimum settings were found, 35.8 g/L of C18:1 non-acetylated acidic SLs was tested. The addition of 0.1% formic acid to both mobile phases was also used to improve protonation of the acidic SLs. A final routine gradient method with the “best” conditions (1.4 ml/min flow rate, 45 °C temperature) was used with a shortened gradient of 30% ACN for 5 min, 30%–41.9% ACN for 8.5 min, 41.9%–100% ACN for 4.15 min, 100% ACN for 10 min, 100%–30% ACN for 1 min, and equilibration at 30% ACN for 10 min for a total run time of 38.65 min.

**Table 1.** Conditions Tested for High-Performance Liquid Chromatography Development.

**Condition**	**Temperature (°C)**	**Flow rate (** **ml** **/min)**
1	30	0.7
2	35	0.7
3	40	0.7
4	30	1.4
5	35	1.4
6	40	1.4
7	45	1.4
8	50	1.4

### LC–MS Structural Congener Confirmation

To confirm the forms and retention time of SL congeners, analysis was performed on a Q Exactive Plus equipped with an electrospray ionization probe coupled to an UltiMate 3000 UHPLC. The optimized conditions from the *Determination of Chromatography Conditions for Peak Resolution* section were applied and the peaks for the C18:1 diacetylated lactonic SL and C18:1 non-acetylated acidic SL standards were analysed and mass confirmed.

Data acquisition was conducted in full scan mode with data-dependent acquisition for the top five most abundant ions per scan. The scan range was 100–1,500 *m*/*z* with a resolution of 70,000 and an automatic gain control target of 3^e6^ and a maximum integration time of 200 ms. The acquisition was conducted in negative and positive ion mode. UV absorbance was monitored using a diode array detector with a scan range of 198–800 nm.

### Analytical Method Comparison

#### Sampling method

To ensure confluent samples were taken for the analytical techniques in this work, the whole volume of the fermentation broth was placed in a 50-ml Falcon tube (Fisher Scientific, UK). The samples were well mixed by vortexing at 2,000 rpm for 30 s until a vortex formed in the centre of the liquid. A sample was quickly retrieved while the broth was still agitated.

#### Liquid:liquid extraction and gravimetric quantification

The LLE quantification method used is identical to that found in Ingham and Winterburn (2022). In brief, well-vortexed samples (5 ml) were taken and heated at 60 °C for 15 min. Hexane extraction was used for rapeseed oil removal and quantification, followed by SL recovery and quantification using ethyl acetate. Equal volumes of hexane were added to the fermentation broth sample and vortexed at 2,000 rpm for 15 s and the hexane phase removed onto a pre-weighed aluminium tray, with the extraction repeated a further two times. The final extraction samples centrifuged at 5,000 rpm for 10 min to ensure full removal of the hexane phase. To remove SL product, three separate ethyl acetate extractions were performed and dispensed on pre-weighed aluminium trays. Both solvent extractions were allowed to dry overnight at ambient temperature on their pre-weighed aluminium tray. These trays were then weighed and the final concentrations calculated in relation to the final volume of broth.

#### Anthrone assay

The anthrone method is based on the work of Buschmann and Wodarczak (1995), with adaptations. The reagent was prepared by adding 200 mg of anthrone in 5 ml pure ethanol at room temperature and brought to a final volume of 100 ml using 75% sulphuric acid and then allowed to cool to ambient temperature.

Fermentation samples were retrieved from the freezer and heated at 60 °C. A 0.5 ml sample was diluted 1 in 150 with pure ethanol and centrifuged at 4,000 rpm for 10 min. A 50 µl resultant supernatant was added to 1,000 µl anthrone reagent and heated at 105 °C in vacuum tubes for 9 min and allowed to cool. The absorbance of the resulting sample was measured at 625 nm, using a blank 1,000 µl anthrone reagent and 50 µl ethanol. Calibration curves of pure glucose (from 1 to 0.001 g/L) and C18:1 diacetylated lactonic SL (from 1 to 0.001 g/L) were prepared under the same conditions. Eleven fermentation flasks that presented varying concentrations of final SL and initial media composition were selected for quantification (see Supplementary Appendix 1). To ascertain the effect of certain media components on the anthrone assay, 50 µl of corn steep liquor (at 5, 0.5, 0.1, and 0.05 g/L), glycerol (at 10, 0.1, 0.02, and 0.01 g/L), and rapeseed oil (25, 50, and 100 µl) were individually tested with the assay.

Final glucose concentrations in the selected broths were quantified with HPLC-RI using the method described in the study by Ingham and Winterburn (2022). In brief, an isocratic mobile phase of 5 mM H_2_SO_4_ was run at 0.6 ml/min with an Aminex HPX-87P column at 50 °C and quantified using a RefractoMax 520 refractive index detector (Thermo Fisher Scientific, UK). The final concentration of glucose in the samples was determined and the contribution of this to the observed anthrone assay response was calculated from the calibration curve.

#### Quantification method comparison

The HPLC and LLE methods were compared using JMP Pro 17 and the Matched Pair Analysis platform to perform a paired *t*-test and the Wilcoxon signed rank test. HPLC and anthrone assay comparisons were made using JMP Pro 17 Graph Builder and the Distribution platform.

## Results

### HPLC Sample Preparation

**Fig. 2.**
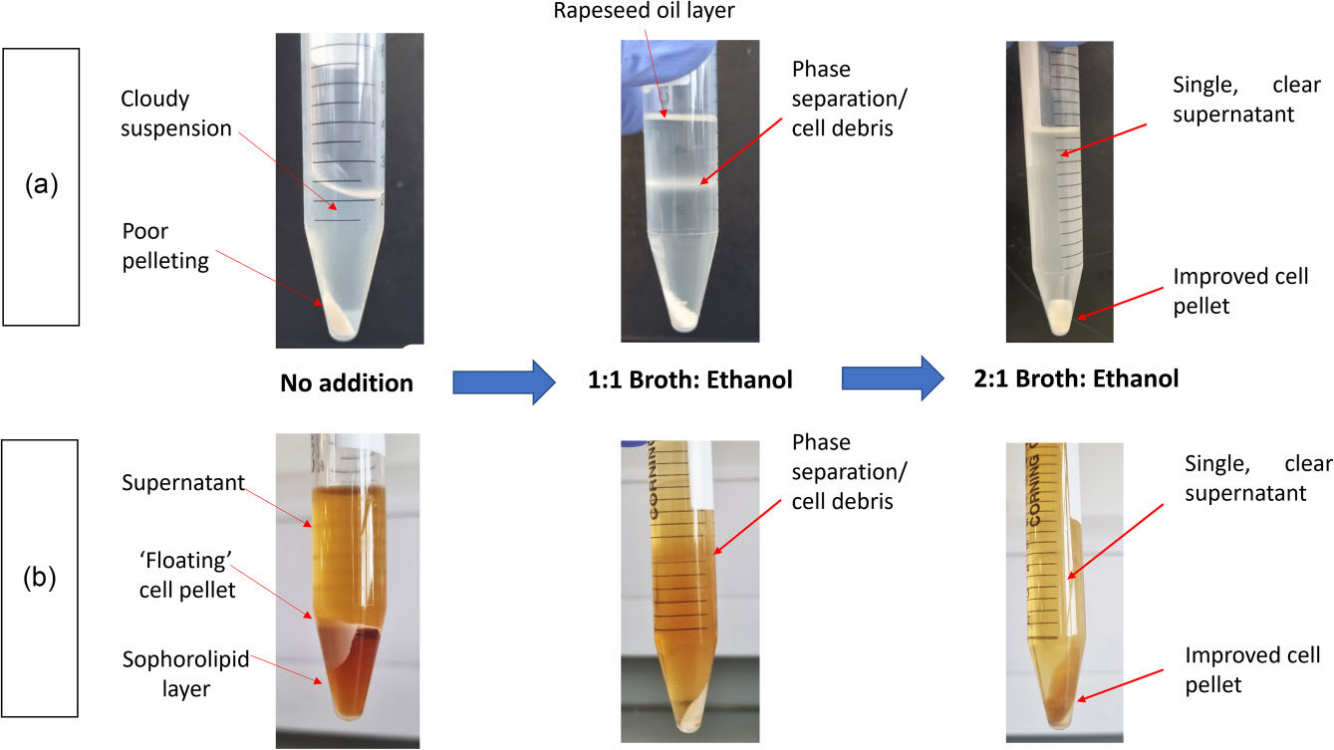
Effect of ethanol addition to the supernatant of two fermentation samples (168-hr fermentation) following centrifugation at 4,000 rpm for 10 min. Ethanol was sequentially added, vortexed (2,000 rpm, 30 s), and centrifuged (4,000 rpm, 10 min) between each step shown.

To prepare samples for HPLC, a confluent, single-phase supernatant with no cell debris is required. As shown in Fig. 2, centrifuged fermentation broths can form either two systems: (1) a four-phase system containing SLs, a floating cell pellet, clear supernatant, and rapeseed oil (bottom, Fig. 2) or (2) a three-phase system containing a “soft” (easily reconstituted) cell pellet, cloudy suspension, and rapeseed oil (top, Fig. 2). To create a single, homogeneous supernatant (containing the water-soluble, lipid-soluble, and SL phases), ethanol was added to the whole broth sample at a 1:1 and 2:1 ratio. The initial addition of 1:1 ethanol:broth improved both the cell pelleting and the clarity of the suspension; however, a “halo” formed between the two phases (see Fig. 2). Analysis of this “halo” with microscopy revealed it to be rich in *Starmerella bombicola* ATCC 22214 cells (see Supplementary Appendix 2). A 2:1 addition of ethanol:broth led to full clarification of the fermentation broth, with a single supernatant containing all of the broth components and a single cell pellet at the bottom of the tube with no separate SL phase (see Fig. 2). To determine whether the cell debris “halo” was related to the media components, a fresh solution of 100 g/L glucose, 100 ml/L rapeseed oil, 5 g/L corn steep liquor, and 4 g/L ammonium sulphate was tested at 1:1 ethanol to see whether the “halo” shown in Fig. 2 appeared. Ethanol addition did not cause any phase separation in the media component samples.

To determine whether the ethanol addition improved the recovery of SLs, a harvested fermentation broth was prepared with/without the final ethanol addition and the supernatant collected, filtered, and tested under the final HPLC conditions (see the *HPLC Condition Adaptations* section for details) quantified for C18:1 diacetylated lactonic SL. As shown in Table 2, the sample from the 2:1 ethanol showed over double the final concentration of C18:1 diacetylated lactonic SL, despite being from the same fermentation sample.

**Table 2.** C18:1 Diacetylated ω − 1 Lactonic Sophorolipid Concentration of Different Sample Preparation Methods Quantified With High-Performance Liquid Chromatography.

**Sample type**	**Relative** **C18:1** **ω − 1 diacetylated lactonic** **concentration (g/L)**	**Dilution adjusted concentration (g/L)**
Supernatant only	14.53 + 0.04	14.53 + 0.04
Supernatant + 2:1 ethanol	10.35 + 0.07	31.05 + 0.21

### HPLC Condition Adaptations

**Fig. 3.**
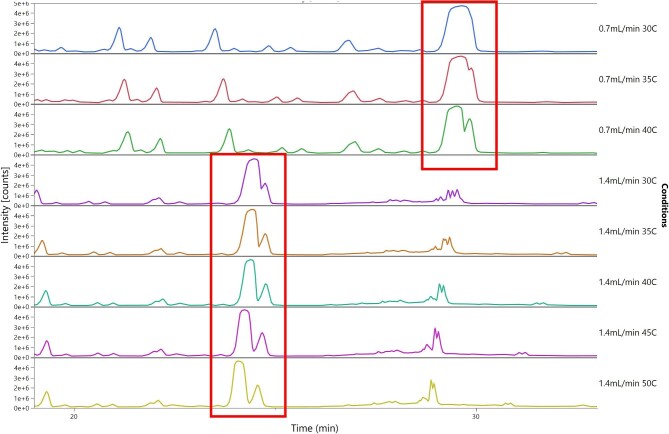
Separation of primary peak forms in C18:1 diacetylated lactonic standards as flow rate and column temperature were altered. Highlighted in red.

During initial characterization of the C18:1 diacetylated lactonic SLs, two isoforms were found in the area where the primary (largest) peaks of the standard are found. In order to properly separate these peaks, the flow rate and temperature were altered, as shown in Fig. 3. Initial attempts to change the temperature at 0.7 ml/min led to the appearance of a secondary peak as the temperature rose above 35 °C; however, the separation was still not sufficient as the peaks were too close together to accurately integrate the peaks and make measurements of the area under the curve. Increasing the flow rate from 0.7 to 1.4 ml/min caused an expected, immediate shift in retention time of the peaks (from 29.4 to 24.7 min) and reduction of peak width, further separating the two peaks. Increasing the temperature at 1.4 ml/min from 30 to 50 °C led to continued improvement in increasing the separation between the two peaks. The highest level of separation was found at 50 °C (0.49-min difference); however, 45 °C was selected due to its suitability to improve the longevity of the column (as it was closer to the 30–40 °C recommended by manufacturer) and the minor difference between the two temperatures (0.46 min vs. 0.49 min difference for 45 or 50 °C, respectively).

**Fig. 4.**
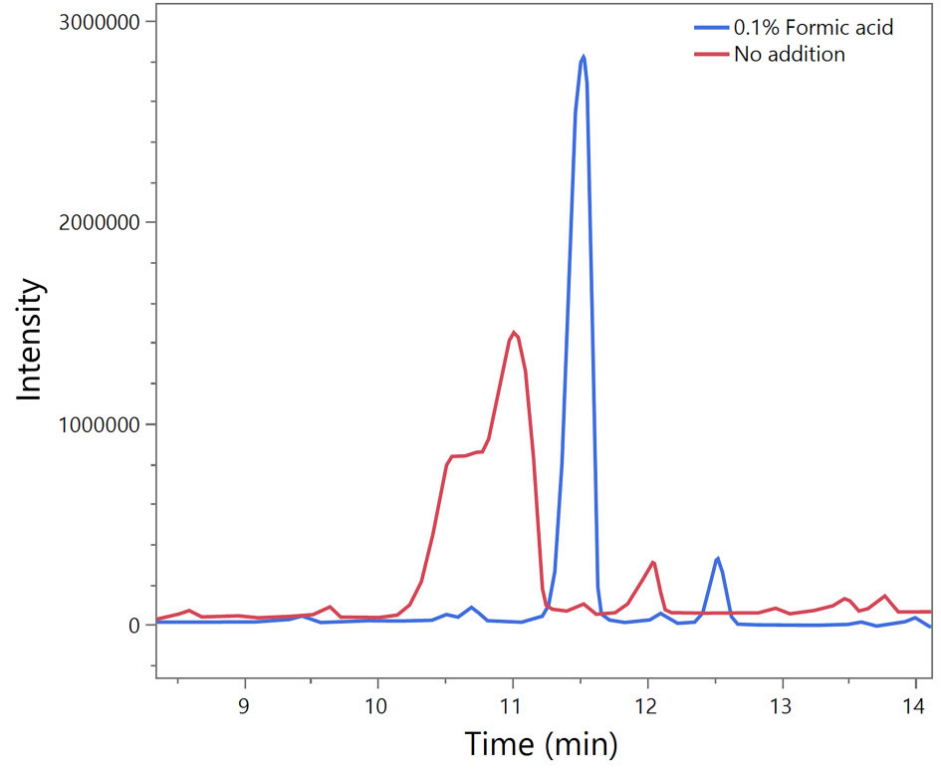
Chromatogram of acid SLs before and after the addition of 0.1% formic acid to the water and acetonitrile mobile phase: 1.4 ml/min flow rate; 45 °C column temperature; and 35.8 g/L C18:1 non-acetylated acidic SL. SL = sophorolipid.

Following the selection of optimized conditions (1.4 ml/min, 45 °C), a standard of C18:1 acidic non-acetylated SL was tested. As shown in Fig. 4, the peak showed significant deformation/tailing, indicative of the presence of a mix of non-protonated and protonated forms. To resolve, 0.1% formic aid was added to both the water and ACN mobile phases. This led to a significantly improved peak shape, with a single, narrow peak.

### Confirmation of SL Congener Structure With LC–MS

To confirm that the peaks identified were correctly the C18:1 diacetylated lactonic and C18:1 non-acetylated acidic forms, LC–MS analysis was performed in positive and negative mode with the optimized conditions found in the *HPLC Condition Adaptations* section. The chromatogram was searched by the expected monoisotopic mass of the peaks and the retention time and peak shape(s) were compared. Peaks of monoisotopic mass 688.3670 and 622.3564 Da were found at the same retention time of the C18:1 diacetylated lactonic and C18:1 non-acetylated peaks, respectively, under the HPLC–UV method, confirming the structure.

### Detection Optimization and Limits

Wavelength scans between 190 and 400 nm were taken with the diode array detector to find the optimum wavelength for the C18:1 diacetylated lactonic and C18:1 non-acetylated acidic forms. Both forms were optimally detected at 198 nm (see Supplementary Appendix 3 and Appendix 4). The detection lower/upper limits (at 198 nm) were found to be 0.3–24 g/L standard for the C18:1 diacetylated lactonic and 0.5–8.75 g/L standard for the C18:1 non-acetylated acidic standards. Sample concentrations above/below these ranges showed non-linearity in calibration curves as the detector became saturated. The robustness of the method was compared by looking at the variation in the peak area from repeated measurements of two C18:1 diacetylated lactonic standards (from Biobase Europe and Biosynth) at different concentrations. Variation was low on repeated measurements confirming the method's reliability, with 3.21% average variation (see Supplementary Appendix 5 and Appendix 6).

### Comparison of LLE and HPLC Method

**Fig. 5.**
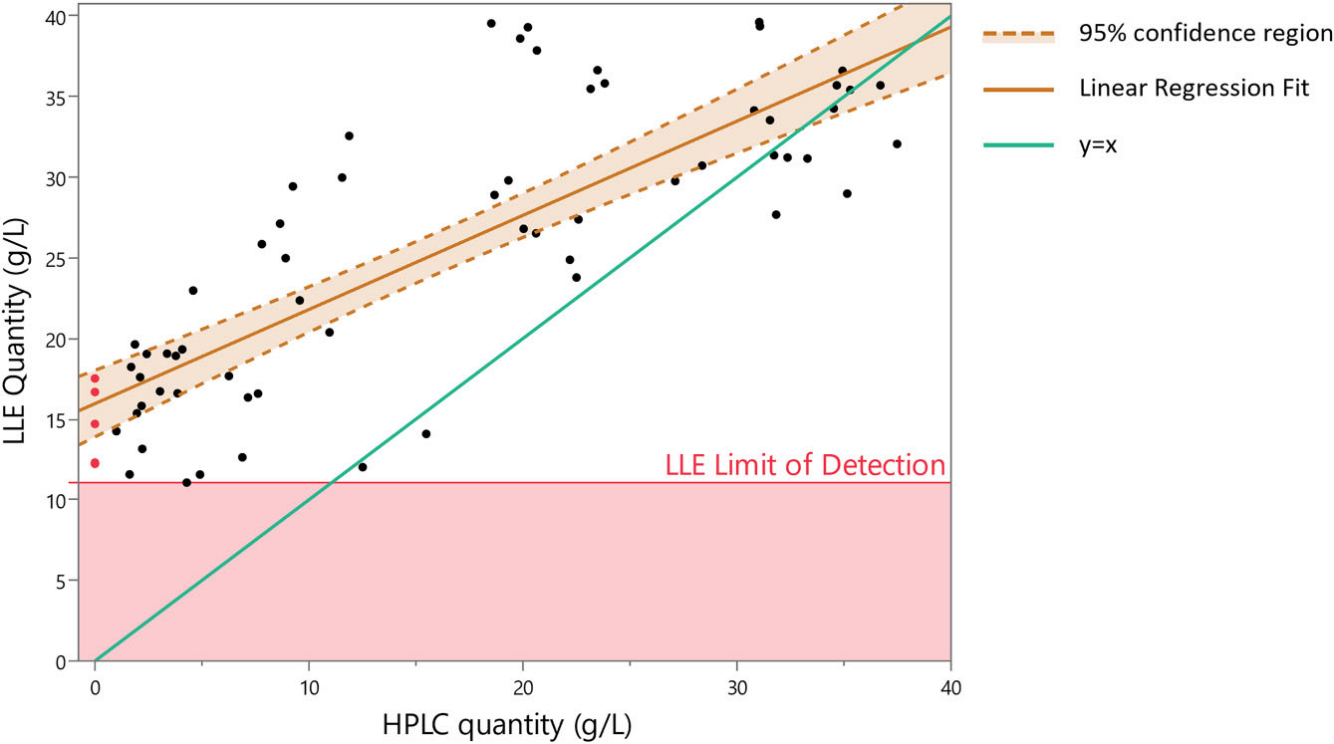
A comparison of the final quantification values between HPLC and LLE, with the LLE limit of detection range shown in red. All samples at 0 g/L (marked red) are below the detection limit of the HPLC method. Quantification methods are compared with a linear regression fit (orange line) with a 95% confidence region (light orange). A *y* = *x* line (green) has been added to allow comparison of the slope of the linear regression fit between the methods at different quantities. HPLC = high-performance liquid chromatography; LLE = liquid:liquid extraction.

To compare analytical methods, the SL content of 75 fermentation broths was quantified with LLE and HPLC, with the latter targeting the subterminal hydroxylated C18:1 diacetylated lactonic SL (as C18:1 non-acetylated acidic SLs were not detected), as shown in Fig. 5. Of the 75 samples, four demonstrated no detectable quantity (under the 0.3 g/L detection limit) of C18:1 diacetylated ω − 1 lactonic peak. Inversely, the LLE method was not capable of detecting below 11.06 g/L, even in flasks where no SL peaks (related to the C18:1 diacetylated lactonic and acidic forms) were detected with HPLC. Some linearity was shown between the two methods, with an *R*^2^ of .658, with both methods capable of generally distinguishing low/high producing flasks; however, certain flasks did not follow this trend, leading to overall low correlation. This is shown well in flasks with LLE quantities close to 40 g/L, which present a range of HPLC quantification values from ≈20 to ≈33 g/L C18:1 diacetylated ω − 1 lactonic SL.

**Fig. 6.**
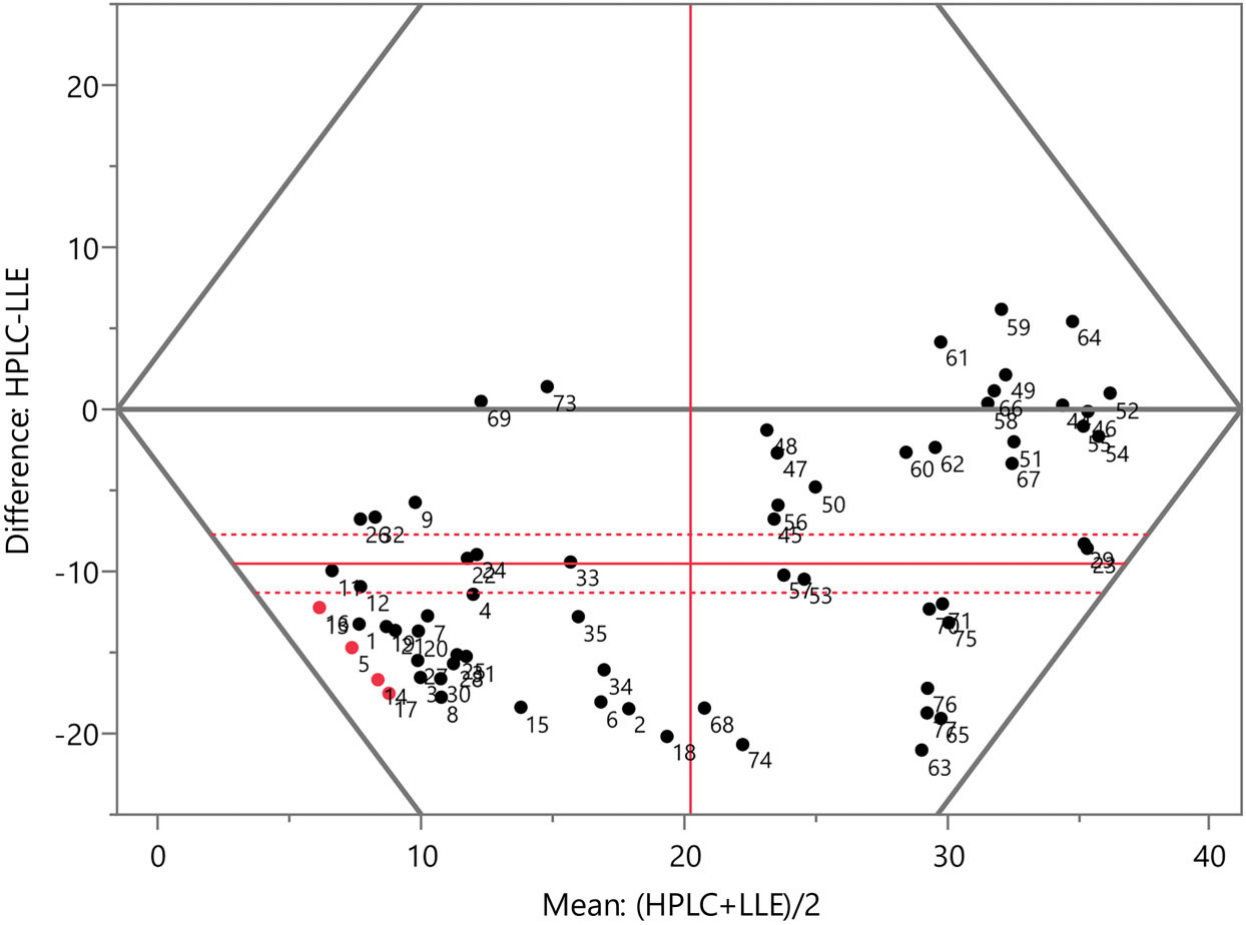
The Tukey mean difference plot with the difference (*y*-axis) and mean (*x*-axis) of the two responses. The upper portion (>0 g/L difference) represents where the LLE value is less than the HPLC, and the lower portion (<0 g/L difference) where it is more. The vertical red line represents the mean of the pairs (mean of means). Points marked in red are those below the HPLC 0.3 g/L quantification limit. Mean difference (horizontal line) with 95% confidence intervals (dotted line). Means of the pairs (vertical line). HPLC = high-performance liquid chromatography; LLE = liquid:liquid extraction.

To test the sample-by-sample differences between the two methods, matched-pairs analysis was performed using standard paired *t*-tests. Firstly, the difference in the estimated SL concentration between the analysis methods was calculated and the distribution across the tested flasks was analysed to see whether the fit was normal. It was found that the matched pair differences fit better to a Normal 2 Mixture than a single Normal distribution (see Supplementary Appendix 7). This could be because the 75 samples are made up of a cluster of low SL content and a second cluster of higher SL content. This non-normality was the reason for performing a Wilcoxon signed rank test alongside the paired *t*-test, but both tests pointed to the same conclusion (see Supplementary Appendix 8) that the difference between the two analytical tests was statistically significant in the two tailed *t*-test (*p* ≤ .0001) and the Wilcoxon signed rank test (*p* >[sum of ranks] = .0001). The Tukey mean difference plot (see Fig. 6) shows that the mean difference (solid red horizontal line) was −8.949 g/L (HPLC–LLE) with a 95% confidence interval of ±1.771 g/L—indicating that LLE results are consistently higher across the range of SL content. The 0-g/L difference line was not contained within the 95% confidence interval, demonstrating a significant difference to at least the 0.05 level. As shown in Fig. 6, there was better agreement (lower difference) in the samples containing higher amounts of SL, as indicated by the clustering along the zero-difference line on the right-hand side of the axis.

### Sensitivity and Accuracy of the Anthrone Assay

**Fig. 7.**
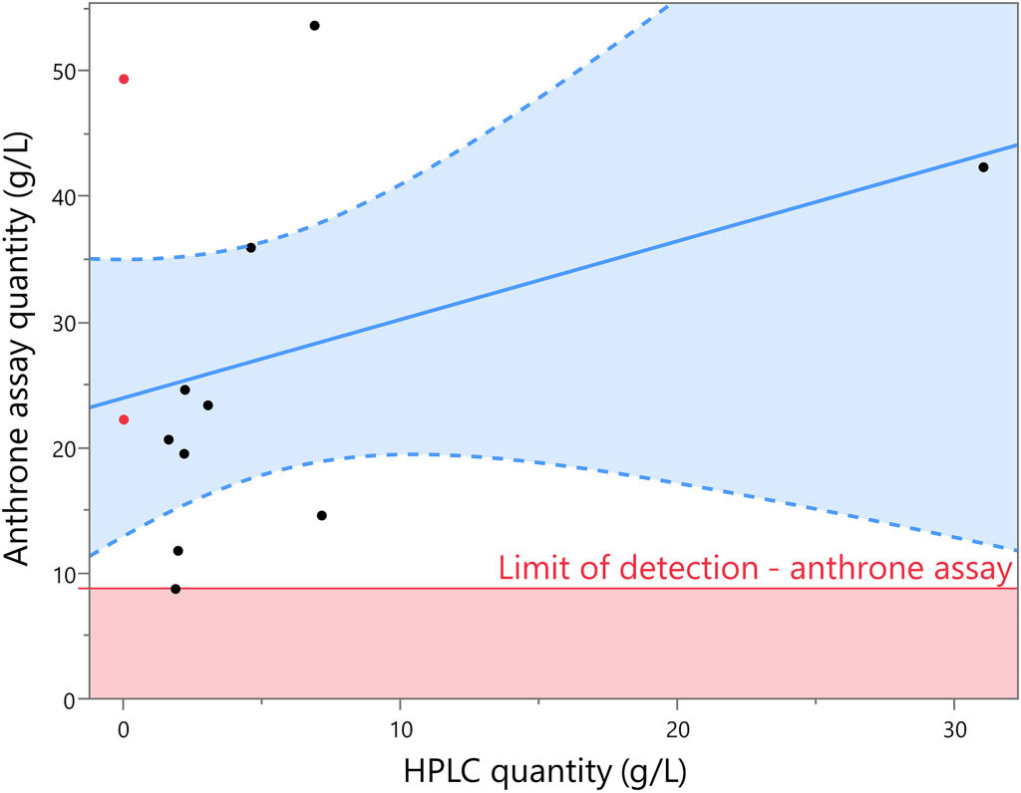
A comparison of the final quantification values between HPLC and anthrone assay, with the anthrone assay limit of detection range shown in red. All samples at 0 g/L (marked red) are below the detection limit of the HPLC method. Quantification methods are compared with a linear regression fit (blue line) with a 95% confidence region (light blue). The anthrone assay values were calculated using a calibration curve from standards of C18:1 diacetylated lactonic sophorolipid. HPLC = high-performance liquid chromatography.

To compare the anthrone assay to the other methods, 11 fermentation samples were selected (see Supplementary Appendix 1) for analysis and compared against the HPLC quantification of C18:1 diacetylated ω − 1 lactonic SL. The values produced from the anthrone assay of SL fermentation samples were compared against a calibration curve of either pure glucose or C18:1 diacetylated lactonic standard, with the latter being used for final comparisons. As shown in Fig. 7, there was no correlation (*R*^2 ^= .129) between the two methods, with the anthrone assay demonstrating a mean difference of 22.0026 g/L (*p* ≥ .0002) from HPLC. The lowest quantity of SLs found with the anthrone assay was 8.736 g/L. The two lowest HPLC quantified samples (<0.3 g/L, marked red in Fig. 7) did not show the lowest quantity with the anthrone method, instead having 22.22 and 49.34 g/L. Further to this, the highest tested HPLC value (31.040 g/L) was not the highest quantity detected with the anthrone assay. Residual glucose in the samples was quantified with HPLC and the equivalent response to the anthrone assay was subtracted from the final SL quantity; however, this led to numerous negative values, as low as −13.68 g/L, so was not used (see Supplementary Appendix 13).

The effect of media components on the anthrone assay was explored, testing glycerol, corn steep liquor, and rapeseed oil at different concentrations. Glycerol (from 0.01 to 10 g/L) had no effect on the final measurement, whereas corn steep liquor contributed an equivalent of 0.1–6.8 g/L to the final SL concentration if present in samples (0.1–5 g/L, see Supplementary Appendix 14). Rapeseed oil addition caused the formation of a dark, black compound that could be absorbed at 625 nm (see Supplementary Appendix 10). The exact contribution of rapeseed oil to any potential anthrone assay measurements could not be determined as the concentrations used were higher than those present in the fermentation samples (i.e. final concentration never exceeded 100 ml/L in samples compared to the 500 ml/L tested here).

## Discussion

### Sample Preparation

In most fermentation processes, the broth is a simple mixture of water-soluble media components and product and an insoluble cell mass that can be easily separated, leaving a single-phase supernatant that can be further characterized. Biosurfactant fermentation broths demonstrate a complex, multiphase composition featuring a lipid, water, cell, and SL phase. This separation of phases creates two key issues: (1) to suitably use HPLC, all of these components (apart from the insoluble cell components) should be dissolved in a single phase that properly represents the relative proportion of a given analyte to the total volume of the fermentation broth and (2) the presence of a free oil/lipid-rich phase quickly fouls the membrane filters (i.e. nylon, Polytetrafluoroethylene) used to prepare HPLC samples for analysis.

A vitally under-discussed aspect of using an oil/water fermentation media is the ability to produce samples that are confluent (representing the relative portion of the oil/water phase found in the fermenter/flask). Without sufficient agitation during sampling, the oil phase quickly separates, especially during the early stages of fermentation where there are high levels of oil and low levels of biosurfactant, with shaking by hand usually being insufficient, even with baffled flasks that aid in the mixing. In directly sampling from bioreactors, this process is simplified by the ability to sample with a high level of agitation provided by the aeration/impeller rotation. However, shake flasks are hard to mix thoroughly without external agitation (i.e. magnetic stirrer bar), which is cumbersome when sampling numerous flasks. This study found success by collecting the whole volume of the shake flask (50 ml) and vortexing in a 50-ml Falcon tube, taking a sample while the broth is still “vortexed” to better gain confluency. However, this method requires the whole broth to be harvested, making time course measurements difficult, and is not suitable for larger scale fermentations as process are developed. No resolution for confluent sampling of shake flasks has been discussed in the literature.

Following sampling, centrifugation is most typically used as the initial stage for clarification of fermentation samples, which allows full separation of the aforementioned phases. However, the position of the SL layer can cause complications as it can appear under the cell pellet, causing the cell layer to “float” above and making recovery of the product of interest difficult (as shown in Fig. 2). This SL phase is typically associated with the lactonic SL forms (particularly those with a higher number of acetyl groups) that show low water solubility when compared to the acidic SL counterpart, which has a free carboxylic acid group (Hu & Ju, 2001; Van Bogaert et al., 2011; Zerkowski et al*.*, 2006). The appearance and position of this phase are associated with the pH of the media and the glucose concentration of the fermentation media, the latter causes the phase to appear on the top or bottom of the fermentation broth, which has been used advantageously in the recovery of the SL phase in downstream processes (Dolman et al*.*, 2017; Van Bogaert et al., 2011). However, the need to obtain a single phase to provide correct proportional quantification is important, as such a suitable “all-in-one” solvent is needed that can form a single analyte phase and distinct cell pellet following centrifugation.

A popular solvent for SL recovery is ethyl acetate as it is able to dissolve both the SL and oil phases. However, this has been found to cause significant issues with cell debris (discussed in more detail below), making sample preparation challenging. Ethanol was chosen as a solvent due to its wide availability, lower cost, and proven solubility for SLs with the hope of it avoiding the cell debris issues found with ethyl acetate (Davila et al*.*, 1993; Hu & Ju, 2001). As shown in Fig. 2, initial addition of ethanol (1:1) improved clarity but led to the formation of a layer of cell debris in the middle of the supernatant and a “weak” cell pellet that easily re-enters the water-soluble phase. This cell debris is similarly found during the LLE process (following the oil phase removal by hexane and the first ethyl acetate addition, see Supplementary Appendix 11) and sits between the water-soluble and ethyl acetate-soluble phases. This phenomenon has not been extensively reported in the literature but is a significant complication/barrier to SL purification and sample preparation. The exact cause is unclear; however, we have found it to be associated with the formation of glycerol >5 g/L (a by-product of triglyceride breakdown in rapeseed oil), and microscopic analysis (see Supplementary Appendix 2) shows it to be a cell-rich phase (Ingham & Winterburn, 2022). Other attempts to “break” this phase by longer/higher levels of centrifugation and “salting out” (addition of increasing concentration of sodium chloride) were unsuccessful. A 2:1 ethanol solution led to removal of the cell debris, full pelleting of the cell mass, and a single, clear supernatant with the oil and SL phases contained within. SLs have been shown to be soluble in ethanol, and the addition of the neutral ethanol likely increased the pH of the solution (from the acidic pH fermentation broth), which is shown to improve solubility (Chen et al*.*, 2020). Similarly, the rapeseed oil fully entered into solution in all 75 fermentation broths tested with HPLC (ranging from 15.4 to 184 ml/L initial concentration). Certain components of the fermentation broth, such as glucose, can show low insolubility in ethanol; however, the final ethanol concentration was low enough (66%) to dissolve them. In cases where other types of plant/vegetable oils to rapeseed oil have been used (not reported here), sequential addition of ethanol (above 2:1 ratio) has achieved full dissolution; however, caution must be made to avoid over-diluting such that the SL of interest can no longer be detected. It is worth noting that acidic SLs do show lower solubility in ethanol and may require higher dilution with ethanol or use of another solvent that can also solve the associated cell debris formation. Choosing the correct solvent is a balance of ease of access, safety, and specificity for dissolving both hydrophilic and hydrophobic compounds. There are reports of methanol showing good solubility of SLs; however, the presence of cell debris has not been mentioned (Samtani et al*.*, 2018).

To evaluate the effect of ethanol addition on the recovery of SLs, a sample with a cloudy broth and no distinct SL phase was tested for its C18:1 diacetylated lactonic concentration (see Fig. 1). The results (see Table 2) show that the addition of ethanol significantly increased the final SL concentration (doubling the final value). It is likely that the ethanol addition improved the recovery by (1) preventing the formation of a separate SL phase “under” the cell pellet during centrifugation that could not be recovered and (2) dissociating SLs associated within the cell pellet and oil phase. This disassociation would also be advantageous by preventing loss during the subsequent filtering process required for HPLC analysis. It should be noted that crystallization can occur in ethanol and has been seen in our standards stored at −20 °C; however, reheating the solution at 60 °C for 30 s with vortexing fully dissolves the crystals (Hu & Ju, 2001). For long-term storage, there have been reports that SLs can be affected when stored outside of a pH of 7.0–7.5, leading to the degradation of acetyl and ester bonds (Van Bogaert et al., 2011). In this study, degradation was not observed in the standards used; however, to ensure the stability of SLs during storage, single-use inset HPLC vials were utilized to prevent degradation due to varying temperatures and potential evaporation following injection.

Correct sample preparation is vital to obtain a confluent sample that, when quantified, represents the actual concentration of the analyte in question. SL production flasks are complicated by the presence of multiple phases that occur in different ratios depending on the fermentation conditions (media, time, productivity) that hinder the quantification process. The application of ethanol in this work created a single-phase supernatant that reduced the appearance of cell debris and improved the recovery of SLs, allowing for more accurate determination of SL concentration in the fermentation broth and hence reliable calculation of key fermentation metrics such as productivity.

### HPLC Method Development

HPLC has become increasingly prevalent in modern laboratories for quantification purposes, driven by decreasing costs and the demand for shorter turnaround times for results. HPLC offers several advantages over other analytical techniques, including precise quantification, high sensitivity, and broad applicability/flexibility in various fields. The separation of SL biosurfactants with HPLC poses a significant challenge due to the presence of closely related congeners that are naturally generated during the fermentation process, influenced by the feedstock, strain, and fermentation conditions. Variations in fatty acid length, saturation, open or closed ring structures (acidic or lactonic), acetylation (non-, mono-, or diacetylated), mono-acetylation position (C6′ or C6″ hexose), and terminal or subterminal hydroxylation lead to a high number of potential congeners. Between fatty acid chain lengths of 16–19 carbons, there are 120 possible congeners that could be created with variation in the aforementioned structural characteristics, not including other rare forms that are possible (Kobayashi et al*.*, 2023; Price et al*.*, 2012). This can lead to congeners with minor differences in their polarity, which are challenging to separate using HPLC. Accurate separation of SL congeners is crucial for understanding their properties, bioactivity, and industrial applications. Differences in physiochemical properties and functionalities among the various congeners highlight the need for precise and reliable analytical methods to achieve baseline separation and to provide confidence when reporting the quantification of a specific congener (Ahn et al., 2016; Koh et al., 2016).

The first reported method for HPLC separation of SLs was from Davila et al*.* (1993), where ELSD was used with a reversed-phase column and gradient elution (ACN:water) to alter the polarity of the mobile phase and separate SL congeners; however, there have been a wide range of HPLC methods reported in the literature, typically using a reversed-phase C18 column with an ACN and water gradient (Hirata et al*.*, 2021; Jiménez-Peñalver et al*.*, 2020; Konishi et al*.*, 2018; Nunez et al*.*, 2001, 2004; Ribeiro et al*.*, 2012). It is worth noting that without matching the exact make/type, HPLC columns can vary widely despite being under the “C18” moniker, with variations within/between manufacturers in carbon load, pore size, surface area, silica type, and packing type, all of which affect the separation, meaning some form of process optimization is usually required to achieve the desired separation and peak quality (Euerby & Petersson, 2003; Žuvela et al*.*, 2019). Method development is often under-reported in literature surrounding SLs, so the work here gives useful insights for those who will have to develop their own methods.

The goal of this study was to use HPLC with a detector type that is commonly found in analytical laboratories (UV) to suitably separate and quantify C18:1 diacetylated lactonic SLs, achieving a stand-alone peak that could easily be quantified (by peak area) without peak crossover. The choice to focus on the separation of the C18:1 diacetylated lactonic congener was made due to the preference of wild-type *S. bombicola* ATCC 22214 to produce lactonic forms (due to the ready conversion of acidic forms to lactonic structures) and use C18:1 fatty acids (Huang et al., 2014; Saerens et al., 2015; Wongsirichot et al., 2021).

Initial trials using a larger particle size C18 column (5 µm; Nucleosil 120-5) were unsuccessful in separating the congeners of C18:1 diacetylated lactonic and C18:1 non-acetylated acidic forms from standards (not shown). This was exacerbated in fermentation broths where co-eluting peaks appeared and made quantification impossible. To improve the separation, an alternative column (Nucleosil 100-3) was chosen with a reduced particle size (5–3 µm) to improve the efficiency of separation, whereas a reduced length (120–100 mm) was chosen to lower the back pressure brought on by reducing the particle size. Initial tests at 0.7-ml/min flow rate and 30 °C column temperature did not show good separation of the C18:1 diacetylated lactonic peaks (see Fig. 3). Alteration of the gradient “steepness” with these conditions did not improve the separation, so the decision was made to explore the alteration of the temperature and flow rate to tackle two issues: the separation of the peaks and the width.

In this work, a combination of altered temperature and flow rate resulted in improved separation efficiency and reduced peak width, leading to a distinct separation between the two lactonic peaks (see Fig. 3). Column temperature, often overlooked in reversed-phase HPLC optimization, influences the retention behaviour of analytes and, subsequently, the selectivity (ability to measure two separate analytes) of the chromatographic method and can be utilized to enhance the separation between closely eluting peaks, while reducing the back pressure on the system (Dolan, 2002). This was clear in Fig. 3, where an increase from 30 to 50 °C allowed the difference between the two peaks to go from 0.28 to 0.49 min (at 1.4-ml/min flow rate).

Increasing flow rates improve the resolution of the chromatography by reducing the peak broadening or peak width; however, care must be taken to find the optimal setting, as the broadening can be influenced by either too low (via longitudinal diffusion) or too high (via mass transfer) flow rate settings (McCalley, 2000). The large peak width of the primary (first in retention time) peak causing the smaller secondary peak to become masked in the chromatogram. By increasing the flow rate from 0.7 to 1.4 ml/min, the peak width of both the primary and secondary peaks was reduced, allowing the two peaks to be more clearly separated and enabling an improved determination of the area under the curve during peak integration. It is important to note that the flow rate did not cause any change to the selectivity of the system; the retention time difference between the two peaks was unaffected by the changes in flow rate (i.e. 0.7–1.4 ml/min at 35 °C has the same separation of 0.2 min etc.). The final operating conditions of 1.4 ml/min and 45 °C were chosen to separate the two peaks.

The two peaks were found to exhibit identical mass spectra despite their difference in retention time, indicating that they were the subterminal (ω − 1) and terminal (ω) hydroxylated C18:1 diacetylated forms of the lactonic SL. As characterized previously by ^1^H-Nuclear Magnetic Resonance, Gas Chromatography-MS, and fast atom bombardment mass spectroscopy, the subterminal (ω − 1) form appears first and is typically the most abundant form made by wild-type strains (Cavalero & Cooper, 2003; Davila et al*.*, 1993; de Koster et al*.*, 1995; Nunez et al*.*, 2001). Similarly, no peaks associated with the hydroxylated forms were found in the quantified fermentation samples. Within the example shown in Fig. 3, complete separation of the subterminal and terminal peaks was not achieved at the optimum settings. However, the experiments were conducted using a 50 g/L standard concentration that exceeded the linear limit of detection of the UV detector (24 g/L), leading to significant peak broadening. Analysis between the linear range (0.3–24 g/L) demonstrated sufficient separation of the two peaks.

Following the optimized separation of the terminal and subterminal C18:1 diacetylated lactonic peaks, the optimum settings were tested on the C18:1 non-acetylated acidic standards. Unfortunately, the conditions used were found to cause significant tailing and distortion of the acidic peak, which was associated with the partial dissolution of the analyte as it exhibited a mix of protonated and deprotonated forms (see Fig. 4). The addition of 0.1% formic acid to each mobile phase to fully protonate the acidic forms resulted in a distinct single peak for C18:1 non-acetylated acidic form. Conversely, this inclusion did decrease the sensitivity (peak height) of the C18:1 diacetylated lactonic form (although sensitivities down to 0.3 g/L were achieved). The inclusion of formic acid is commonplace in chromatographic methods and eases the method transfer to LC–MS where it is required for improved ionization, but methods focusing solely on lactonic form quantification with UV may want to choose to omit it or replace with an acid with better UV transparency, such as *ortho*-phosphoric acid. The need to include the formic acid in the method, despite its effect on the sensitivity for lactonic forms, highlights the challenge in dealing with a family of compounds that have such a wide array of physiochemical characteristics. Another consideration for transfer to LC–MS is the ∼1-ml/min flow rate limit for MS units—the 1.4-ml/min flow rate may overwhelm the evaporation process of the mass spectroscope and lead to condensation and poor ionization. To avoid this, flow splitters can be used to reduce the incoming flow from the HPLC unit.

As mentioned, SLs were first detected with ELSD, which has been widely accepted as the “gold standard” for characterizing and quantifying SL congeners due to its ability to detect congeners without the need for specific functionalities such as ionization (for MS) or absorbent functional groups (for UV). ELSD operates by evaporating the HPLC eluent and detecting the resulting dry particles, meaning its only requirement is that analyte does not evaporate, which can be achieved by adjusting the conditions of the ELSD to maintain a temperature well below the boiling point of the analyte in question. However, ELSD units are separate, nonstandard components for HPLC that come at additional capital and operational cost, meaning it is not commonly found in analytical laboratories. This is compounded by higher running costs (nitrogen supplies, additional maintenance) and complexity when compared to the standard UV method, for example, narrow linear dynamic ranges and the larger degree of detector optimization required to gain sufficient resolution of the target peaks. Conversely, UV detection has widespread availability as an integrated component in most HPLC systems and was chosen for this study to demonstrate its applicability where ELSD may not be available. The UV detector was able to linearly detect up to 24 g/L lactonic diacetylated C18:1 and 8.75 g/L acidic non-acetylated SLs. The peak response was observed at a wavelength of 198 nm for both forms, which would benefit a UV detection system that could only measure one wavelength per sample run. Interestingly, these findings contradict the suggestions made by previous studies, which have proposed wavelengths of 207 and 210 nm that were found to be significantly smaller in response in this study (Alfian et al*.*, 2022; Thaniyavarn et al*.*, 2008; Wadekar et al*.*, 2012). Improper selection of the UV wavelength can lead to lower overall sensitivity of the method, so the use of diode arrays is invaluable in finding optimum wavelengths.

While HPLC provides a method that can selectively isolate any given SL congener for quantification, it is limited by the commercial availability of analytical-grade standards. As of writing, the only commercially available standards are those of C18:1 diacetylated lactonic, C18:1 mono-acetylated acidic, and C18:1 non-acetylated acidic forms. It is possible to prepare standards for in-house use, but this requires substantial material and the development of separatory methods such as preparative HPLC, which is a significant undertaking and a likely reason why commercially available standards are so limited.

A suitable quantification method for C18:1 diacetylated lactonic and C18:1 non-acetylated acidic forms was developed that was capable of separating the subterminal and terminal C18:1 diacetylated lactonic forms, allowing for accurate and representative quantities to be reported. This method was further applied for the quantification of SLs in 75 total fermentation samples, with a focus on the subterminal C18:1 diacetylated lactonic SL, and was used as the “reference” method to provide a measure of the true quantity of SLs in the fermentation samples.

### Application and Limitations of LLE

LLE is a commonly used tool in the selective isolation of key components for quantification across a range of industries/areas. Most typically hexane:ethyl acetate extraction is used to clean and isolate SLs from the fermentation broth, wherein hexane is used to remove residual lipid substrate whereas ethyl acetate extracts the SL product. The application of hexane for oil recovery has been commonplace in industrial oil recovery for years, whereas ethyl acetate has been used for the recovery of glycolipid biosurfactants from as early as the 1970s (Jones & Howe, 1968; Liu & Mamidipally, 2005; Lohani et al., 2015; Rau et al., 2001; Zerhusen et al., 2020). However, both solvents are non-specific and do not provide a sole measurement of the substrate or product in question. For ethyl acetate, there has been little research into the specificity for the recovery of biosurfactants/SLs in fermentation broths.

The 75 fermentation broths tested with HPLC were quantified with LLE and the results compared. As shown in Fig. 5, the LLE method was not capable of accurately quantifying the amount of SLs present in the fermentation broths. Through the matched-pairs analysis (see Fig. 6), it was possible to demonstrate the difference statistically, with a difference in both the *t* (*p* ≤ .0001, two tailed) and Wilcoxon signed rank (*p* ≤ .0001) tests. The average mean difference between measurements was −8.949 g/L (HPLC vs. LLE), meaning the LLE method consistently over-quantified the amount of SLs present in the sample. This is shown well by the lowest quantifiable amount being 11.06 g/L; even when little C18:1 diacetylated lactonic SL is present in the sample, the LLE method is not capable of distinguishing between a non-productive flask and a productive flask at lower concentrations of the final product. This limitation is particularly detrimental in development stages where screening of feedstocks, fermentation conditions, and strains relies on an accurate comparative measurement of the final product. It is possible that other structural variants of SLs outside of the targeted C18:1 diacetylated lactonic form could be contributing to the LLE measurement; however, these were not detected (either due to lack of radiance to UV absorption or being present in quantities below the lower detection limit) in the HPLC method.

The specific components that are co-extracted with the SLs are not known; however, the likely components are in either the initial media constituents that are added to the broth or components that are created over the course of the fermentation (by the organism or through chemical reactions). One such component could be via the oil substrate and the limitations of the hexane extraction process. Hexane extraction is intended to remove all “non-SL components” to ensure the ethyl acetate recovered components are representative of the final fermentation SL concentrations. However, evaluation of hexane extraction efficiency revealed that it was unable to recover the entire initial amount of rapeseed oil added. In a series of sequential washings using equal volumes of hexane in a fermentation broth mixture containing 100 ml/L rapeseed oil, the recovery efficiency was found to be approximately 86.6% (±5.13%) of the actual weight of rapeseed oil initially added (see Supplementary Appendix 12). Consequently, this means there are residual components of the oil that can be subsequently taken up during the ethyl acetate extraction steps. Hexane is incapable of extracting the more polar components of oil including monoacylglycerol and diacylglycerols, whereas recovery of saturated and short-chain (<C10) fatty acids is poor (Lalman & Bagley, 2004; Li et al*.*, 2014; Lopes & Bernardo-Gil, 2005; Rashid et al*.*, 2018). Given that the fatty acid sources used for SL production are usually plant-derived sources that are rich in fatty acids of differing carbon length and saturation and form, it is likely that oil-based components are co-extracted with the SLs in the ethyl acetate step. Interestingly, the largest degree of difference between the two quantification measurements was found with high nitrogen (>2.5 g/L cornsteep liquor (CSL) and >2 g/L ammonium sulphate); however, it is hard to discern whether this difference is caused by the low SL production (as nitrogen inhibits production) and the minimum 10 g/L quantification limit or by a biomass-associated component (as more nitrogen creates greater biomass). One consideration is that unproductive fermentation runs have lower utilization of the oils supplied, including the lower chain length fatty acids that would normally be utilized in de novo fatty acid synthesis by *S. bombicola*, leading to a greater proportion of oil impurities that are eventually extracted by the ethyl acetate phase (Van Bogaert et al., 2011). However, this does not explain how there are LLE quantifications with high levels (up to 40 g/L) and lower quantification via HPLC (∼20 g/L), such as those shown in Fig. 5. The initial media composition in these flasks is well distributed between the concentrations of glucose, rapeseed oil, and CSL/ammonium sulphate, although there may be some additional components being produced aside from SLs that are co-eluted. Possible investigations with untargeted LC–MS studies may be able to better discern what is co-eluted in the LLE method.

Another area to consider with potential co-extraction from feedstocks is from “alternative” or non-food-grade feeds. In the study by Ingham et al*.* (2023), it was found that unknown compounds from lignocellulosic-derived sugar feeds were co-extracted in the ethyl acetate and hexane phases, leading to large overestimations of the quantities of final rapeseed oil and SLs. While efforts were made to account for this co-extraction by performing hexane/ethyl acetate extracts of the pure feedstocks to calculate crossover, this was only partly effective. As mentioned in that work, the transition to low-cost industrial residues/by-products, crops, and wastes of varying composition will lead to complications in the recovery steps when using solvents, which means efforts should be made to account for any co-elution (if possible) or new quantification methods should be made.

Overall, SL quantification through LLE is a hands-on and lengthy laboratory process that can be complicated by minimum volume requirements that can be difficult to perform when phases do not separate as expected. Despite this, the method is capable of crudely isolating SLs for further analysis and has a lot of value at scale where the goal is recovery and not accurate quantification. Similarly, there is no simple alternative to hexane extraction for estimating the proportion of oil that is consumed. While gas chromatography can provide a quantification of fatty acids, it requires methylation of the fatty acids with chloroform/chloromethane that can be hazardous/lengthy.

### Application and Limitations of Anthrone Assay

The anthrone assay is a widely used colorimetric method for quantifying carbohydrates, employing a heated acid medium to convert hexoses into 5-HMF, which subsequently reacts with anthrone to form a green-to-blue colouration that can be measured at a specific wavelength (625 nm). For SLs, the acid/heated conditions lead to the cleavage of the structure’s glycosidic bonds, causing the release of two glucose molecules, acetyl groups (in the case of mono- or diacetylated forms), and a free hydroxylated fatty acid, with the glucose molecules subsequently undergoing formation to 5-HMF (Chatterjee et al*.*, 2022).

Preparation of a calibration curve can be done with either SLs or glucose, with the response from glucose theoretically representing half of the potential response from SLs (due to the two potential glucose molecules released from the sophorose). In this study, calibration curves from both lactonic SL standard and glucose were made. However, the SLs did not generate a response twice as large as that of glucose as expected, with a response 1.61 times greater with SLs. This is likely due to the SL standard not being a “pure” preparation of a single SL congener, with other non-hexose-containing components accounting for a proportion of the test material. Inversely, it is easier to acquire/prepare a “pure” glucose calibration curve, so this may be the preferred route for quantification.

A major limitation of the anthrone assay is that it will react with any free glucose present in the media, which is the primary hydrophilic carbon source used in SL fermentations. Typically, glucose is quantified separately in the samples and the proportional response is subtracted from the final value to gain a closer estimate of the final SL concentration. This was attempted in this work; however, this approach resulted in negative final SL quantities (see Supplementary Appendix 13). Clearly, other components aside from glucose were causing a cross-reaction and overestimation within the anthrone assay. This is shown well in the quantification of corn steep liquor, a commonly used nitrogen source, which contributed up to 6.8 g/L to the final estimated SL values at higher concentrations. Surprisingly, rapeseed oil also reacted to form a dark/black substance that absorbed at 625 nm, possibly from a by-product produced during the hydrolysis of the triglycerides and fatty acids. These contributions of media components to the anthrone reaction demonstrate the non-specificity of the assay and the potential for overestimation of the final SL concentration. Alongside hexoses, the assay causes the formation of furfural from pentoses, which also cause an identical colouration and detection at the same wavelength as 5-HMF. This complicates matters when working with complex/sustainable sugar media, particularly those derived from lignocellulosic biomass, that contain a rich mixture of different mono- and polysaccharides that will hydrolyse to form furfurals in the anthrone assay. These lignocellulosic feeds themselves can be rich in furfural and 5-HMF as a result of their production and can see a further increase in furfural concentrations when autoclaved, as thermal degradation causes the release of 5-HMF (Sugisawa & Sudo, 1969; Woo et al*.*, 2015), further compounding these limitations.

Practically, use of the anthrone assay requires a significant sample dilution to stay within the detection limits of the assay, which is at microgram/milligram quantities of glucose (Leyva et al*.*, 2008; Turula et al*.*, 2010). In fermentation samples where residual levels of glucose can be high (>100 g/L), this dilution inadvertently introduces error, reducing the confidence in the final quantification. The method is labour-intensive and time-consuming and requires the handling of a hazardous assay reagent composed of 75% sulphuric acid. Although methods employing microplates and automated liquid handling have been developed to improve efficiency, they require specialized equipment to operate (Leyva et al*.*, 2008; Turula et al*.*, 2010). Overall, the anthrone assay is incapable of providing accurate quantification of SLs and is likely to overestimate concentrations due to the presence of other reactive materials in the fermentation broth.

## Conclusion

Ultimately, analytical tools are used to quantify SLs to aid the understanding of another system that is being optimized, be that through process optimization, strain screening, feedstock selection, or more. As such, the analytical techniques are often undervalued and poorly understood in terms of their limitations. This effect bleeds into the research, making it difficult to compare enhancements due to the use of different, poorly understood analytical techniques, the data from which cannot be directly compared.

Three techniques are commonly used and were selected for analysis in this study. HPLC is considered the “gold standard” for quantification and, with adjustment of the operating conditions, can isolate and quantify specific SL congeners. Other methods are more generalized, instead offering a total sum of SLs that should still be capable of distinguishing high or low levels of production. The reason for choosing any given method is a mixture of availability, costs, and skill sets to maintain equipment and train users appropriately, meaning no single method will ever become “standard”; however, understanding those limitations and advantages will provide greater confidence when reporting on the quantity of SL produced. As shown, HPLC is an excellent, specific technique that can be applied with UV detection for the C18:1 diacetylated lactonic and C18:1 non-acetylated acidic forms but suffers from the limited number of commercially available standards and the additional costs for detectors, such as ELSD, capable of detecting other congeners that are not UV responsive. LLE is a common choice and shows some linearity with the HPLC method but is non-specific in its extraction, leading to co-extraction of other compounds from the fermentation broth and overestimation of the final SL quantity. Efforts can be made to try to account for this by performing LLE on each media component to understand the co-extraction, but increases hands-on time. The anthrone assay is a cumbersome and inaccurate method that is incapable of accurately quantifying SLs, with low linearity to the HPLC method and, given the hazardous chemicals and complexity, it is recommended that LLE be used if HPLC is unavailable.

## Supplementary Material

kuae021_Supplemental_File
