# Supplementary material for: Determining the accuracy and suitability of common analytical techniques for sophorolipid biosurfactants
Source: J Ind Microbiol Biotechnol. 2024 Jun 21;51:kuae021. doi: 10.1093/jimb/kuae021 (PMC11223654; doi:10.1093/jimb/kuae021)
Supplement: kuae021_Supplemental_File [file kuae021_supplemental_file.docx]

## Determining the accuracy and suitability of common analytical techniques for sophorolipid biosurfactants.

Benjamin Ingham^1^, Rehana Sung^2^, Phil Kay^3^, Katherine Hollywood^2^, Phavit Wongsirichot^1^, Alistair Veitch^4^, James Winterburn^1^*

^1^ Department of Chemical Engineering, The University of Manchester, Oxford Road, Manchester, M13 9PL, United Kingdom

^2^ Manchester Institute of Biotechnology, Department of Chemistry, University of Manchester, Manchester, M1 7DN, United Kingdom

^3^ JMP Statistical Discovery LLC, Wittington House, Henley Road, Medmenham, Marlow SL7 2EB, UK

^4^ Holiferm Ltd., Unit 15, Severnside Trading Estate, Textilose Rd, Trafford Park, Stretford, Manchester, M17 1WA, United Kingdom

* Corresponding author

Email: james.winterburn@manchester.ac.uk, Phone: +44 161 529 3013

## Supplementary Information

| Design | Block | Pattern | Glucose (g/L) | Oil (mL/L) | Nitrogen (g/L) |
| --- | --- | --- | --- | --- | --- |
| 1 | 1 | −−+ | 50 | 50 | 7.5 |
| 1 | 1 | ++− | 150 | 150 | 2.5 |
| 1 | 1 | −++ | 50 | 150 | 7.5 |
| 1 | 1 | −−− | 50 | 50 | 2.5 |
| 1 | 1 | +−+ | 150 | 50 | 7.5 |
| 1 | 1 | −+− | 50 | 150 | 2.5 |
| 1 | 1 | −−− | 50 | 50 | 2.5 |
| 1 | 1* | 000 | 100 | 100 | 5 |
| 1 | 1* | +−− | 150 | 50 | 2.5 |
| 1 | 1* | −++ | 50 | 150 | 7.5 |
| 1 | 1* | +−− | 150 | 50 | 2.5 |
| 1 | 1* | −−+ | 50 | 50 | 7.5 |
| 1 | 1* | +−+ | 150 | 50 | 7.5 |
| 1 | 1* | −+− | 50 | 150 | 2.5 |
| 1 | 1 | +++ | 150 | 150 | 7.5 |
| 1 | 1 | +++ | 150 | 150 | 7.5 |
| 1 | 1 | ++− | 150 | 150 | 2.5 |
| 1 | 2* | 00A | 100 | 100 | 9.2 |
| 1 | 2* | 0A0 | 100 | 184.1 | 5 |
| 1 | 2* | 00A | 100 | 100 | 9.2 |
| 1 | 2* | a00 | 15.9 | 100 | 5 |
| 1 | 2* | 00a | 100 | 100 | 0.8 |
| 1 | 2 | a00 | 15.9 | 100 | 5 |
| 1 | 2 | 0A0 | 100 | 184.1 | 5 |
| 1 | 2 | 0a0 | 100 | 15.9 | 5 |
| 1 | 2 | A00 | 184.1 | 100 | 5 |
| 1 | 2 | 000 | 100 | 100 | 5 |
| 1 | 2 | 00a | 100 | 100 | 0.8 |
| 1 | 2 | A00 | 184.1 | 100 | 5 |
| 1 | 2 | 000 | 100 | 100 | 5 |
| 1 | 2 | 0a0 | 100 | 15.9 | 5 |
| 1 | 2 | +−− | 150 | 50 | 2.5 |
| 1 | 2 | ++− | 150 | 150 | 2.5 |
| 1 | 2 | −+− | 50 | 150 | 2.5 |
| 2 | 4 | 000 | 32.5 | 100 | 0.8 |
| 2 | 4 | +−+ | 50 | 50 | 1.2 |
| 2 | 4 | +−− | 50 | 50 | 0.4 |
| 2 | 4 | −−+ | 15 | 50 | 1.2 |
| 2 | 4 | −−+ | 15 | 50 | 1.2 |
| 2 | 4 | −+− | 15 | 150 | 0.4 |
| 2 | 4 | +−+ | 50 | 50 | 1.2 |
| 2 | 4 | 000 | 32.5 | 100 | 0.8 |
| 2 | 4 | +−− | 50 | 50 | 0.4 |
| 2 | 4 | +++ | 50 | 150 | 1.2 |
| 2 | 4 | ++− | 50 | 150 | 0.4 |
| 2 | 4 | ++− | 50 | 150 | 0.4 |
| 2 | 4 | −++ | 15 | 150 | 1.2 |
| 2 | 4 | +++ | 50 | 150 | 1.2 |
| 2 | 4 | −+− | 15 | 150 | 0.4 |
| 2 | 4 | −−− | 15 | 50 | 0.4 |
| 2 | 4 | −++ | 15 | 150 | 1.2 |
| 2 | 4 | −−− | 15 | 50 | 0.4 |
| 2 | 5 | a00 | 3.0686 | 100 | 0.8 |
| 2 | 5 | A00 | 61.9314 | 100 | 0.8 |
| 2 | 5 | 00a | 32.5 | 100 | 0.1273 |
| 2 | 5 | A00 | 61.9314 | 100 | 0.8 |
| 2 | 5 | 00a | 32.5 | 100 | 0.1273 |
| 2 | 5 | a00 | 3.0686 | 100 | 0.8 |
| 2 | 5 | 00A | 32.5 | 100 | 1.4727 |
| 2 | 5 | 0a0 | 32.5 | 15.9 | 0.8 |
| 2 | 5 | 0A0 | 32.5 | 184.1 | 0.8 |
| 2 | 5 | 0A0 | 32.5 | 184.1 | 0.8 |
| 2 | 5 | 0a0 | 32.5 | 15.9 | 0.8 |
| 2 | 5 | 00A | 32.5 | 100 | 1.4727 |
| 2 | 5 | 000 | 32.5 | 100 | 0.8 |
| 2 | 5 | 000 | 50 | 100 | 0.8 |
| 2 | 5 | 000 | 50 | 100 | 0.8 |

Appendix 1: List of flasks tested with the quantification methods. Those marked with asterisks were tested with the anthrone method.


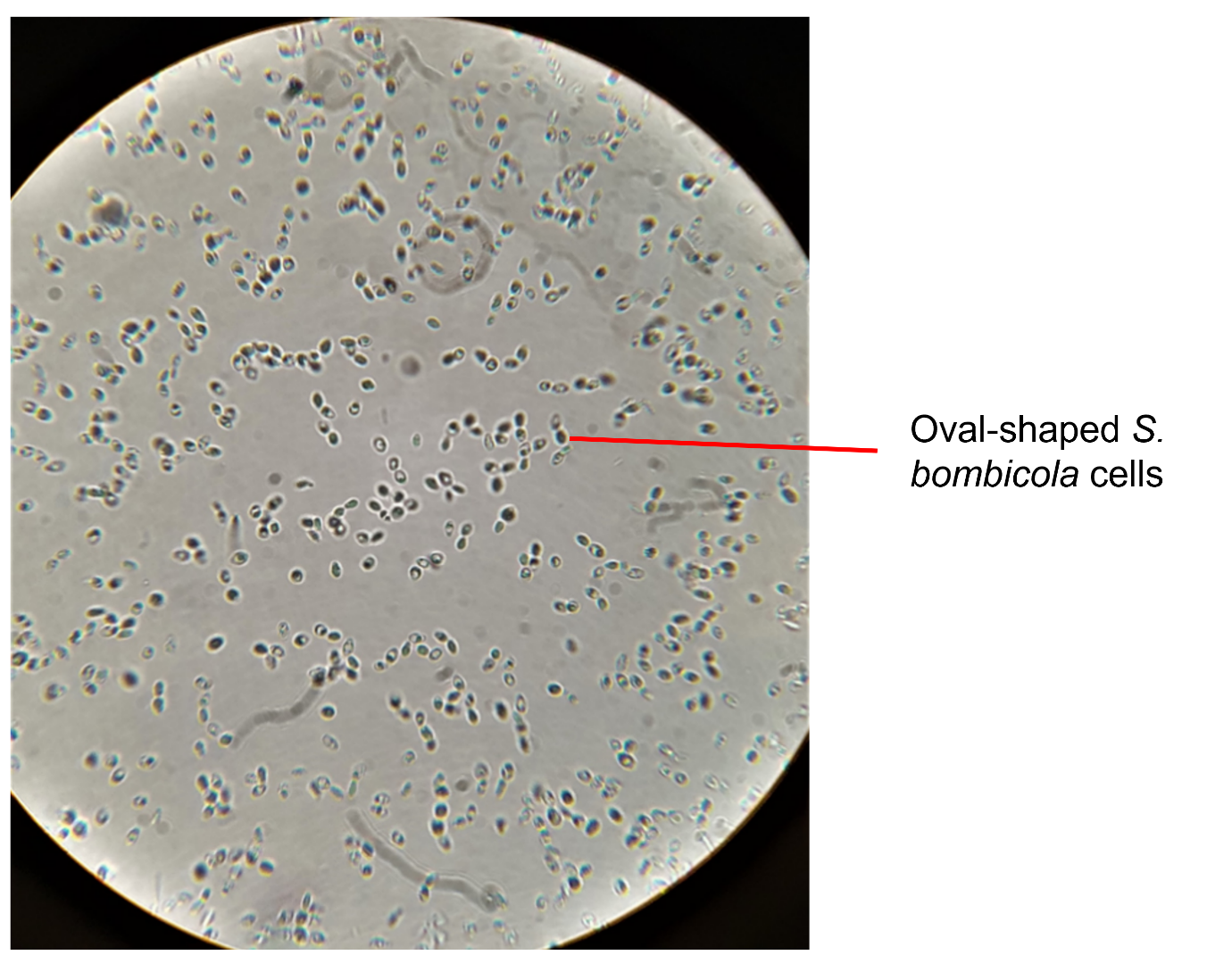


Appendix 2: Light microscopy (100x, Oil immersion) of the 'debris' phase found during the addition of 1:1 ethanol:fermentation broth.


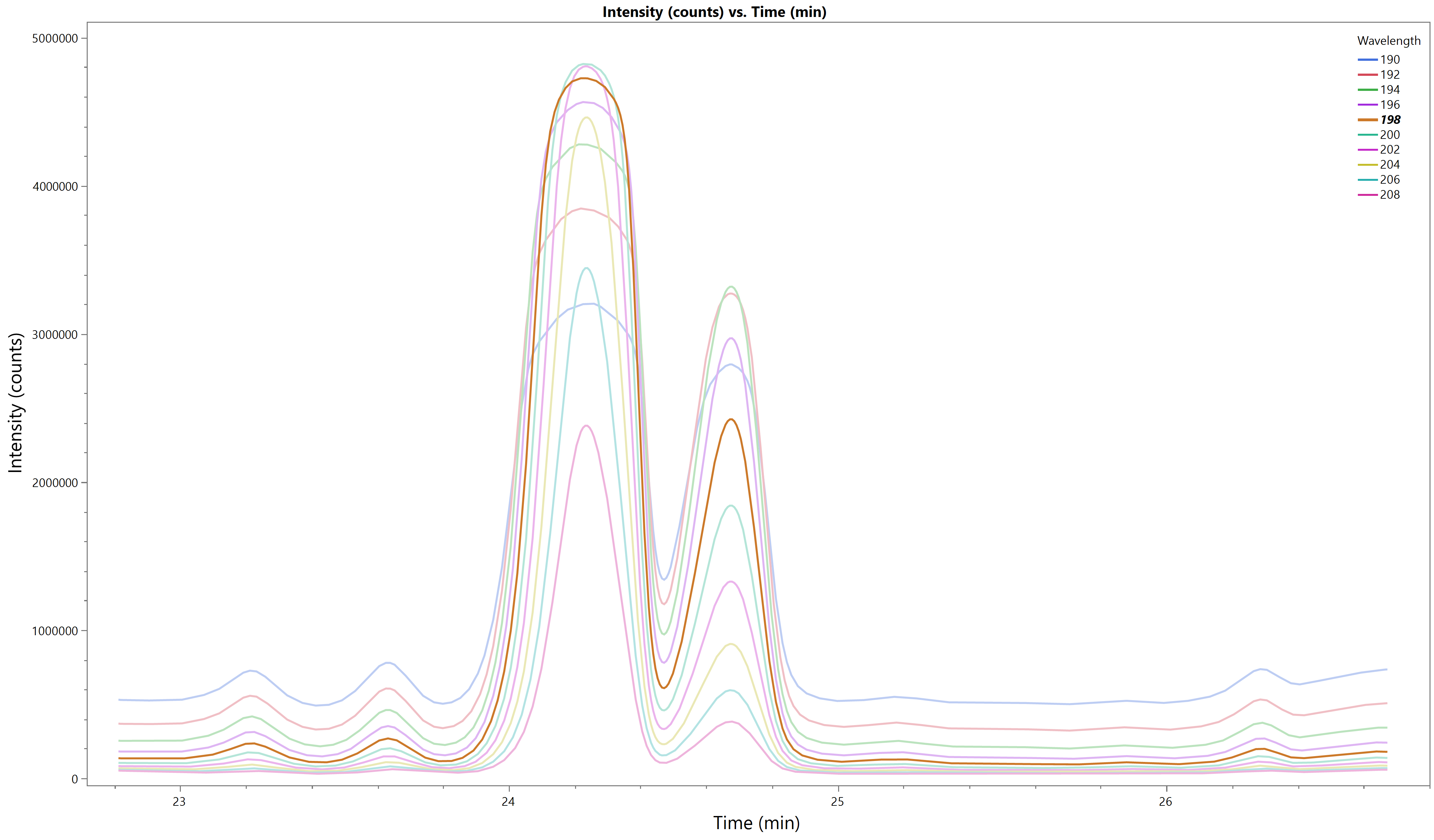


Appendix 3: Wavelength scans of the C18:1 diacetylated lactonic SL peaks from the optimised HPLC method (1.4 mL/min, 45 °C).


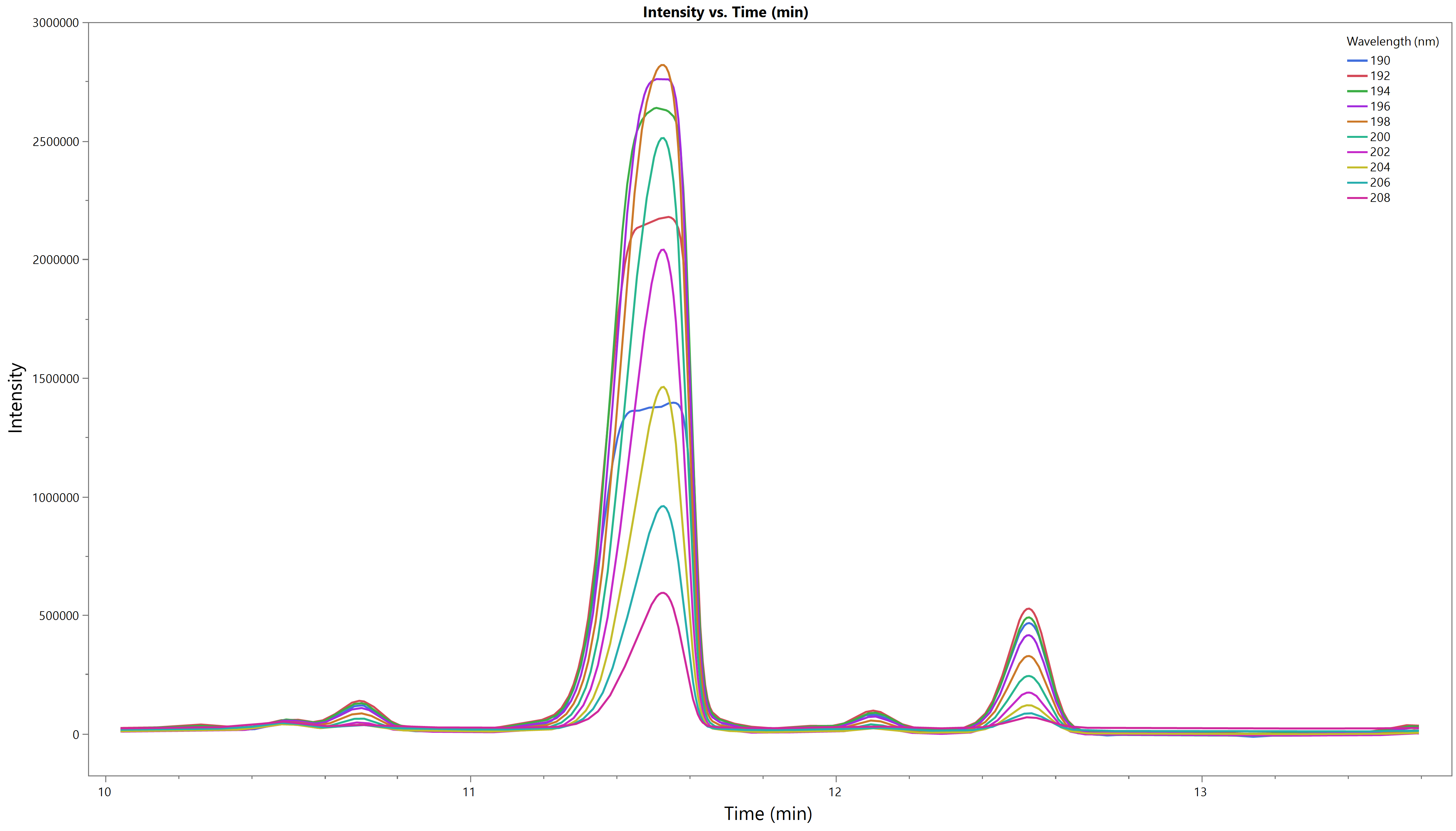


Appendix 4: Wavelength scans of the C18:1 nonacetylated acidic SL peaks from the optimised HPLC method (1.4 mL/min, 45 °C)

Appendix 5: Reproducibility of the HPLC method on 4 tested concentrations of C18:1 diacetylated lactonic SL standard from Biobase Europe. Based on the peak area of the 1st/primary peak (sub-terminal).

Appendix 6: Reproducibility of the HPLC method on 4 tested concentrations of C18:1 diacetylated lactonic SL standard from Biosynth Carbosynth. Based on the peak area of the 1st/primary peak (sub-terminal).


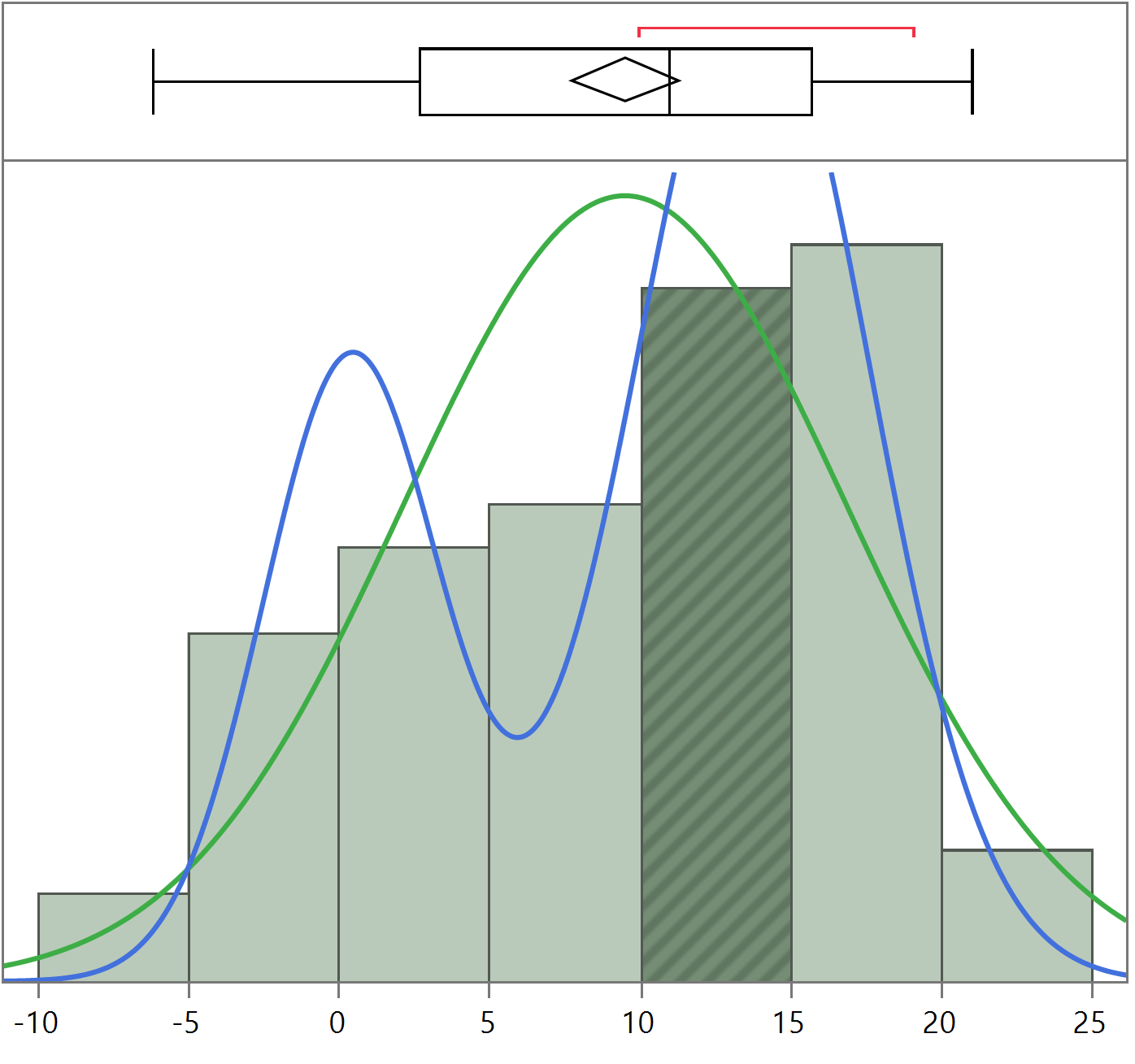


Appendix 7: Distribution analysis of the difference in quantification between HPLC and LLE method. The distribution types are Normal 2 Mixture (Blue, AIC = 516.23102) or Normal (Green, AIC = 522.12099)

| HPLC mean (g/L) | 17.0709 |
| --- | --- |
| LLE (g/L) | 26.0201 |
| Mean Difference (g/L) | -8.9492 |
| Std Error | 0.88871 |
| Upper 95% (g/L) | -7.1784 |
| Lower 95% (g/L) | -10.72 |
| N | 75 |
| Correlation | 0.81261 |
| t-Ratio | -10.0699 |
| DF | 74 |
| Prob > \|t\| (two tailed) | <0.0001 |
| Prob > t (one sided upper) | 1 |
| Prob < t (one sided lower) | <0.0001 |

Appendix 8: Matched pair t-test analysis result for HPLC vs. LLE.

| HPLC | 5.2125 |
| --- | --- |
| Anthrone quantification (SL standard) g/L | 27.2151 |
| Mean Difference (g/L) | -22.003 |
| Std Error | 4.06685 |
| Upper 95% (g/L) | -13.052 |
| Lower 95% (g/L) | -30.954 |
| N | 12 |
| Correlation | 0.35875 |
| t-Ratio | -5.41023 |
| DF | 11 |
| Prob > \|t\| (two tailed) | 0.0002 |
| Prob > t (one sided upper) | 0.9999 |
| Prob < t (one sided lower) | 0.0001 |

Appendix 9: Matched pair t-test analysis comparison between the HPLC and anthrone assay. The anthrone assay is calculated using a calibration curve of C18:1 diacetylated lactonic standard.


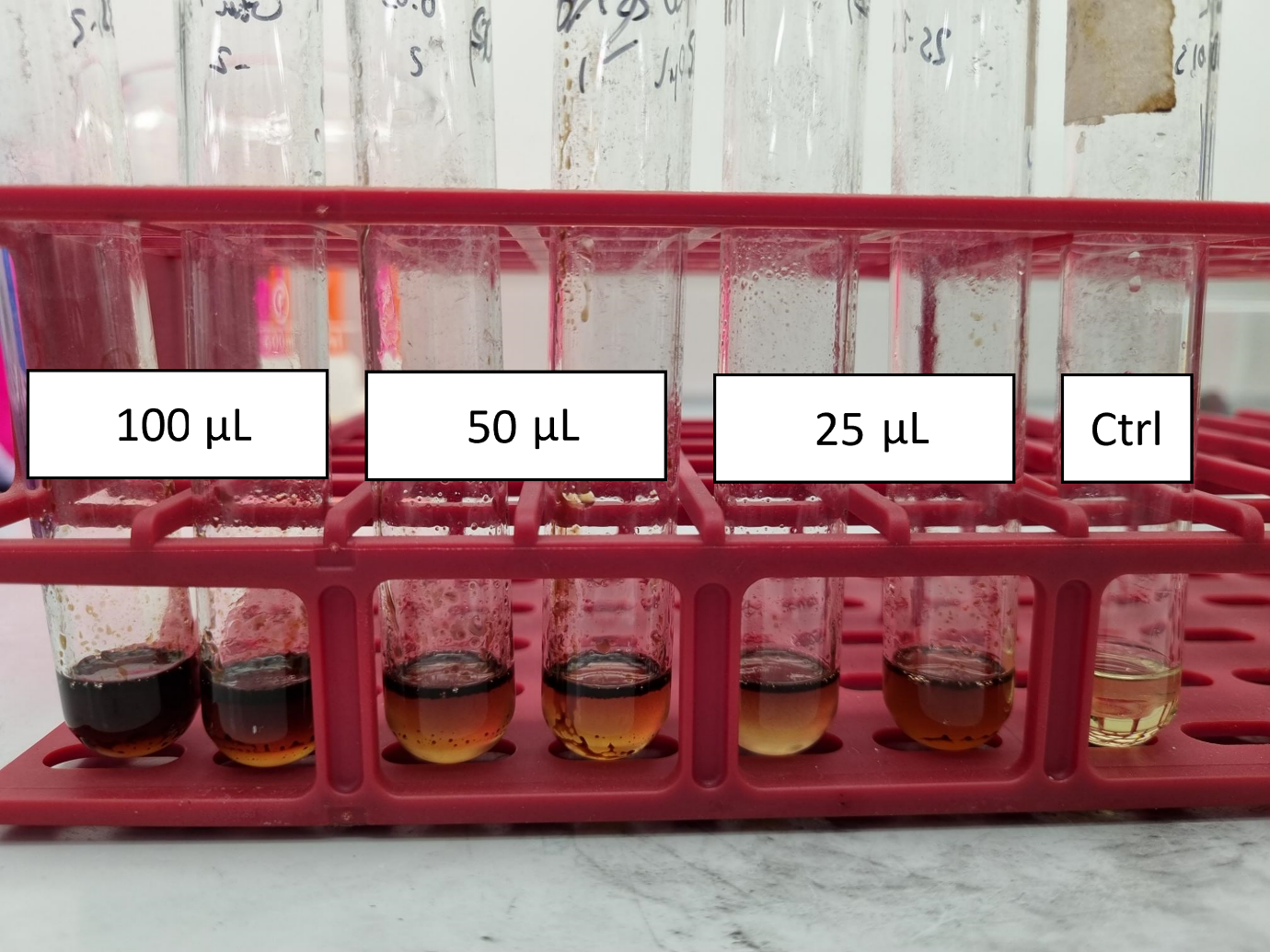


Appendix 10: Effect of different rapeseed oil volumes in the reaction of the anthrone assay.


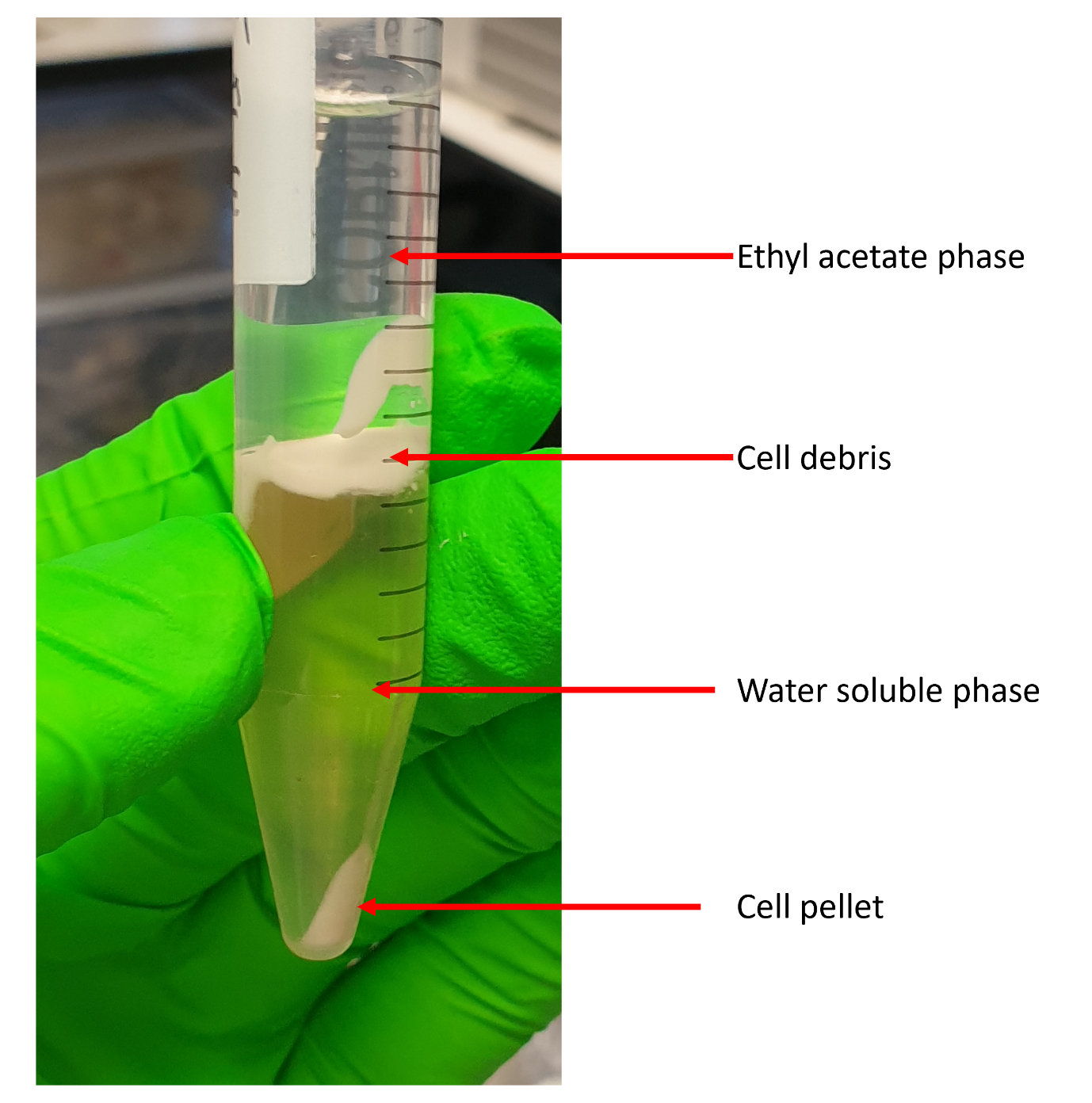


Appendix 11: An example of the cell debris formation following LLE with hexane and ethyl acetate. The sample shown has had duplicate hexane and triplicate ethyl acetate extractions performed. Centrifuged at 4000rpm, 10 minutes.

Appendix 12: Extraction of rapeseed oil from prepared fermentation media using hexane . 5 mL rapeseed oil was dispensed onto a tray and weighed (‘Control’), and a separate 5 mL was placed in fermentation media (100mL/L rapeseed oil, 100 g/L glucose, 5 g/L Cornsteep liquor, 4 g/L ammonium sulfate) and washed with equimolar hexane, with each was being dried separately and weighed. The accumulative weight (‘Total’) was then calculated.

| **Design-Pattern** | **Anthrone quantification (glucose standard) g/L** | **Anthrone quantification (SL standard) g/L** | **Glucose at 168h (g/L)** | **Equivalent response with anthrone (g/L)** | **Final SL quantity (g/L)** |
| --- | --- | --- | --- | --- | --- |
| 1/000 | 17.85 | 8.74 | 19.36 | 14.60 | -5.86 |
| 1/+−− | 83.91 | 53.60 | 18.94 | 14.28 | 39.31 |
| 1/−++ | 35.40 | 20.63 | 23.76 | 17.91 | 2.72 |
| 1/+−− | 41.67 | 24.59 | 38.85 | 29.29 | -4.70 |
| 1/−−+ | 74.69 | 49.34 | 43.49 | 32.78 | 16.56 |
| 1/+−+ | 38.50 | 22.22 | 19.78 | 14.92 | 7.30 |
| 1/−+− | 56.77 | 35.91 | 35.44 | 26.72 | 9.19 |
| 1/00A | 24.57 | 11.78 | 33.76 | 25.45 | -13.68 |
| 1/0A0 | 40.03 | 23.36 | 10.00 | 7.55 | 15.81 |
| 1/00A | 34.87 | 19.50 | 10.00 | 7.55 | 11.95 |
| 1/a00 | 28.32 | 14.59 | 10.00 | 7.55 | 7.04 |
| 1/00a | 65.32 | 42.32 | 10.00 | 7.55 | 34.77102 |

Appendix 13: Selected fermentation flasks for analysis with the anthrone assay.


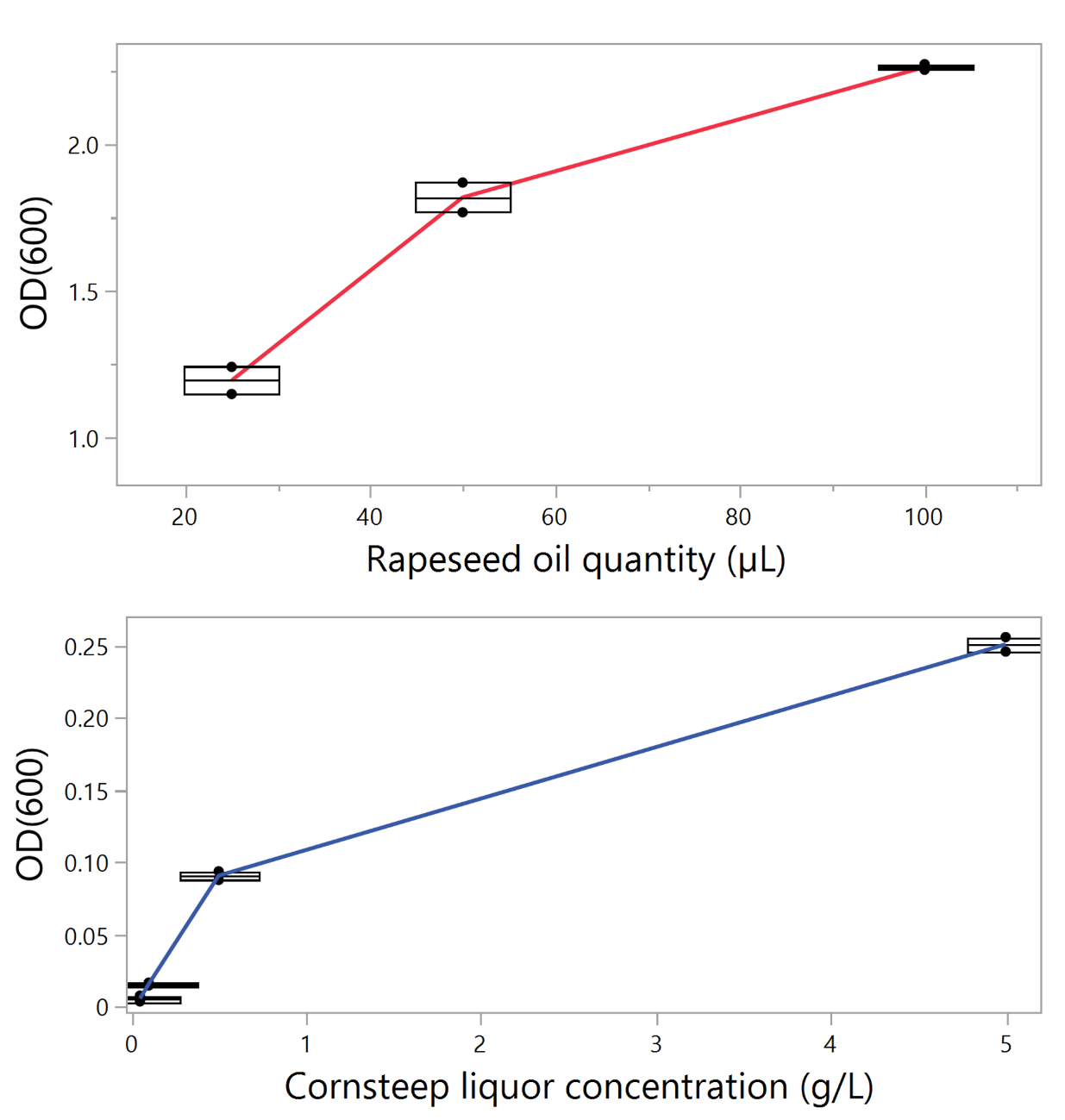


Appendix 14: Effect of cornsteep liquor on the anthrone assay. Spectrophotometric measurement at 620 nm.
